# 1st Global Consensus for Clinical Guidelines for the Rehabilitation of the Edentulous Maxilla: A Single‐Round Survey on Standard, Short, and Zygomatic Implant‐Supported Prostheses

**DOI:** 10.1111/clr.70015

**Published:** 2026-02-24

**Authors:** Franz J. Strauss, Giulia Brunello, Daniel S. Thoma, Ina Kopp, Charlotte Stilwell, Ronald E. Jung, Hom‐Lay Wang, Frank Schwarz

**Affiliations:** ^1^ Center of Dental Medicine, Clinic of Reconstructive Dentistry University of Zurich Zurich Switzerland; ^2^ Universidad Autonoma de Chile Santiago Chile; ^3^ Department of Oral Surgery University Hospital of Düsseldorf Düsseldorf Germany; ^4^ Department of Orthodontics and Dentofacial Orthopaedics, Charité‐Universitätsmedizin Berlin, Corporate Member of Freie Universität Berlin Humboldt‐Universität Zu Berlin Berlin Germany; ^5^ AWMF‐Institut für Medizinisches Wissensmanagement Philipps‐Universität Marburg Marburg Germany; ^6^ Division of Gerodontology and Removable Prosthodontics, University Clinics of Dental Medicine University of Geneva Geneva Switzerland; ^7^ Department of Periodontics and Oral Medicine University of Michigan School of Dentistry Ann Arbor Michigan USA; ^8^ Department of Oral Surgery, Implantology and Oral Medicine Goethe University Frankfurt am Main Germany

**Keywords:** dental implants, questionnaire, short implants, survey, zygomatic implants

## Abstract

**Objectives:**

To gather expert opinions on the use of standard, short, and/or zygomatic implants supporting fixed and removable prostheses in the edentulous maxilla. The aim of this survey was to inform a consensus development process for the subsequent establishment of a clinical practice guideline for the treatment of the edentulous maxilla.

**Materials and Methods:**

A questionnaire was developed in preparation for the 1st Global Consensus for Clinical Guidelines for the rehabilitation of the edentulous maxilla. The survey was conducted in a single round using an anonymous questionnaire. The survey included 19 multiple‐choice questions and 110 statements, where respondents were asked to rank their agreement for each option provided using a 7‐point Likert scale. Consensus was defined as > 75% and ≤ 95% agreement or disagreement, and strong consensus as > 95% agreement or disagreement.

**Results:**

A total of 199 experts, including prosthodontists, oral surgeons, periodontists, and general practitioners from 37 countries were invited to participate. Of these, 118 completed the questionnaire, yielding a response rate of 59.3%. Of the 110 Likert‐scale statements, consensus was reached for 68 (61%) while strong consensus was reached for 5 (4.5%). The statements that achieved strong consensus (> 95%) were: (1) the need for specialized training in the placement of zygomatic implants, (2) the routine use of CT/CBCT imaging for implant planning, (3) the necessity of evaluating maxillary sinus status before performing a lateral sinus lift or (4) when sinus pathology is suspected, and (5) the inclusion of surgical complications as a clinician‐reported outcome in future studies.

**Conclusion:**

The expert survey evaluated treatment approaches for rehabilitation of the edentulous maxilla using standard, short, and/or zygomatic implants to inform a consensus development process and support the establishment of a clinical practice guideline for managing edentulous maxilla.

## 1 Introduction

Complete edentulism and extensive tooth loss remain a major global health concern, with an estimated prevalence of 353 million cases (range: 301–416 million) and an incidence of 26.5 million cases (range: 22.6–31.2) in 2021 (GBD 2021 Diseases and Injuries Collaborators 2024). This debilitating condition negatively affects both oral and general health (Al‐Rafee 2020), contributing to malnutrition from impaired chewing function, communication difficulties, and lower self‐esteem and overall well‐being (Fagundes et al. 2024). Therefore, restoring chewing function and aesthetics is fundamental to improving the quality of life of edentulous patients.

Since the introduction of implant dentistry (Branemark et al. 1977), new protocols have been developed to restore edentulous jaws using implant‐supported prostheses (Messias et al. 2023). However, the optimal treatment approach for the edentulous maxilla remains a topic of debate.

A previous consensus on the management of extraction sockets and the timing of implant placement highlighted that implant therapy protocols are heavily influenced by the clinician's expertise and preferences (Tonetti et al. 2019). Implant‐supported fixed full‐arch prostheses, however, differ significantly in terms of invasiveness and clinical complexity, often requiring more advanced skills (Khoury and Hanser 2019; Urban et al. 2019) compared to a prosthesis for single‐tooth implants in partially edentulous patients (Yao et al. 2018). The treatment options for full‐arch restorations generally vary in the number, size, and location of the implants used. These include standard‐length implants, shorter dental implants (< 8 mm), and zygomatic implants, all of which utilize the available bone following tooth extraction (Jung et al. 2018).

A single‐round survey was conducted involving an international, interdisciplinary group of experts as part of the 1st Global Consensus for Clinical Guidelines (GCCG) 2025. This initiative, titled “Patient‐Centered Clinical Workflow in Implant Dentistry,” aims to establish a consensus on the rehabilitation of the edentulous maxilla. The goal was to gather expert opinions on the use standard, short and/or zygomatic implants supporting fixed and removable prostheses in the edentulous maxilla. The findings were contextualized with existing evidence to guide the consensus development process and identify areas where further systematic reviews or research are needed.

The current summary presents the survey responses regarding maxillary rehabilitations supported by standard, short (< 8 mm), and/or zygomatic implants.

## 2 Materials and Methods

The protocol of this study was approved by the Ethics Committee of the University of Düsseldorf (Protocol no. 2024‐2973). This study was conducted and reported following the “Good practice in the conduct and reporting of survey research” criteria (Kelley et al. 2003).

### 2.1 Study Design

This investigation was conducted as a single‐round survey study in preparation for the 1st GCCG workshop, scheduled for June 2025 in Boston, USA. Its goal was to examine emerging trends and advancements in the rehabilitation of the edentulous maxilla while addressing gaps between common clinical practices and scientific evidence. By collecting expert opinions, the study aimed to provide a realistic overview of current implant treatment strategies, identify areas not covered by existing literature, and highlight discrepancies between expert perspectives and available evidence.

The findings of this survey provided the foundation for developing key questions to be explored through additional systematic reviews or direct discussions at the GCCG workshop in Boston. The Scientific Chairs of the GCCG (Frank Schwarz and Hom‐Lay Wang), supported by the Survey Panel (Giulia Brunello and Todd Schoenbaum) and the Cross‐disciplinary Expert and Patient Panel (Franz‐Josef Strauss and Guo‐Hao Lin), finalized the questionnaire and oversaw the selection and invitation of the experts.

### 2.2 Questionnaire

The expert questionnaire was developed based on the current literature, focusing on the rehabilitation of the edentulous maxilla using standard, short (< 8 mm), and/or zygomatic implants. The initial draft was prepared through the joint efforts of the Scientific Chairs and the Survey Panel.

To ensure clarity and relevance, the questionnaire underwent an iterative validation process, involving multiple rounds of feedback and revisions. In addition to the Scientific Chairs, contributors included the Survey Panel and the Cross‐disciplinary Expert and Patient Panel, as well as leading authors of systematic reviews being prepared for Group 2 of the GCCG (Daniel S. Thoma and UI‐Won Jung). Their input helped refine the questionnaire before obtaining ethical approval.

The questionnaire was created in English and designed to be completed in approximately 20–30 min. The final version, consisting of 40 items (Appendix S1), was organized as follows:

- declaration of consent (item 1);
- professional specialization and working environment (items 2–3);
- familiarity with zygomatic implants (items 4–5);
- short implants versus standard implants (item 6);
- planning phase (items 7–10);
- guided surgery (item 11);
- soft tissue augmentation (item 35);
- timing of implant placement and loading (items 12, 20–21);
- treatment options (items 13–14);
- factors influencing selection of treatment procedures (items 37–38);
- antibiotic prescription in relation to implant placement (items 15–19);
- maintenance (items 22–31);
- home care and occlusal guards (items 32–34);
- factors affecting patient satisfaction (item 36);
- fundamental outcomes to be included in future studies (items 39–40).

### 2.3 Sample Size, Expert Selection, and Data Collection

**FIGURE 1:**
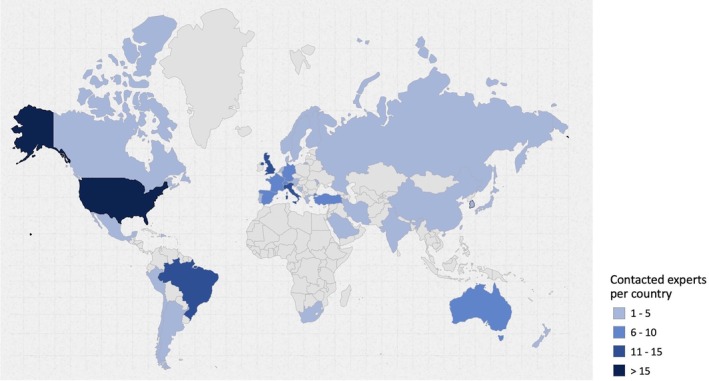
Distribution by country of the contacted experts. Map created using the open source online tool available at https://www.amcharts.com/ (amCharts, Neringa, Lithuania).

There is no established formula for calculating the number of participants needed for an expert survey study, and expectations are based on COMET Initiative guidelines (Williamson et al. 2017) and previous literature (Alarcon et al. 2021; Madianos et al. 2016; Sanz et al. 2019). Therefore, a target sample size of 100 experts was deemed sufficient. Based on an expected response rate of 50%, 199 experts from 37 countries were invited to participate. A detailed breakdown of the experts by country is provided in Table 1 and Figure 1. Because the data were collected anonymously, it was not possible to distinguish between respondents and non‐respondents. The European Association for Osseointegration (EAO) Office distributed the survey link via email to experts selected and approved by the Scientific Task Force. Each organization had discretion over the expert selection process, which included members from leading international implant dentistry organizations, such as the Academy of Osseointegration (AO), the EAO, the International Team for Implantology (ITI), and the Osteology Foundation (OF).

**TABLE 1:** Details and distribution by country of the contacted experts.

Country	No.	%
Argentina	1	0.5
Australia	8	4.0
Austria	4	2.0
Bosnia and Herzegovina	1	0.5
Brazil	13	6.5
Canada	5	2.5
Chile	3	1.5
China	5	2.5
Denmark	1	0.5
Dominican Republic	1	0.5
Finland	1	0.5
France	9	4.5
Germany	8	4.0
Greece	1	0.5
India	4	2.0
Iran	1	0.5
Italy	15	7.5
Japan	2	1.0
Korea (Republic of)	4	2.0
Mexico	2	1.0
Moldova	1	0.5
Netherlands	4	2.0
New Zealand	1	0.5
Norway	1	0.5
Peru	1	0.5
Portugal	3	1.5
Russia	2	1.0
Saudi Arabia	1	0.5
Slovenia	1	0.5
South Africa	3	1.5
Spain	8	4.0
Sweden	3	1.5
Switzerland	9	4.5
Taiwan	1	0.5
Turkey	6	3.0
United Kingdom	15	7.5
United States of America	50	25.1

The survey was launched on October 3, 2024, and remained open for responses until October 25, 2024 (3 weeks). A reminder was sent on October 19, 2024. Participation was voluntary and uncompensated. The survey was administered electronically via Microsoft Forms (Redmond, WA, USA). Informed consent was obtained as the first survey question; participants who did not provide consent were automatically excluded. All data were collected, stored, and processed anonymously.

### 2.4 Agreement Definition

While no universally accepted method exists for defining consensus (von der Gracht 2012), this study applied the following thresholds for 7‐point Likert scale questions:

- *Strong consensus*: achieved when > 95% of the experts “somewhat agreed,” “agreed,” or “strongly agreed” with the statement made, or alternatively when > 95% “somewhat disagreed,” “disagreed,” and “strongly disagreed”;
- *Consensus*: defined when either agreement or disagreement was greater than 75% and no more than 95%;
- *No consensus*: defined when either agreement or disagreement was 75% or less.

For multiple‐choice questions allowing multiple responses, strong consensus for any single option was defined as selection by more than 95% of experts, consensus as selection by more than 75% and no more than 95%, and no consensus as 75% or less (Diamond et al. 2014).

### 2.5 Statistical Analysis

Each question response was analyzed individually using descriptive statistics, with results presented as percentages. Additionally, the degree of agreement for each question was reported as the median score and interquartile range (IQR) (section 1: Appendix S2), following the RAND guidelines (Khodyakov et al. 2023).

In the graphs, the percentage of agreement (%) was calculated by summing the responses “somewhat agree,” “agree,” and “strongly agree.” If the sum of “somewhat disagree,” “disagree,” and “strongly disagree” was higher, that percentage was reported instead, with clarification provided in the figure footnotes. Summary graphs displaying the median and IQR were created following the RAND guidelines and presented in the supplement (Khodyakov et al. 2023).

All analyses were performed using Microsoft Forms, STATA v18, and GraphPad Prism v10.

## 3 Results

Of the 199 experts invited, 123 accessed the survey and 118 provided consent and completed the questionnaire, resulting in a response rate of 59.3%.

### 3.1 Professional Specialization and Working Environment

Most experts held one (76.3%) or multiple (20.3%) specialization degrees, while only four (3.4%) identified as general practitioners. Prosthodontists made up the largest group (43.2%), followed by periodontists (25.4%), oral surgeons (22.9%), and oral and maxillofacial surgeons (18.6%). A small group (8.5%) reported other specialties, mainly implantology.

Regarding working environments, 68.6% worked in private clinics, 54.2% in universities, 11.0% in public hospitals, and 1.7% in other settings. Two thirds (66.1%) worked in only one setting: 47 in private clinics, 29 in universities, 2 in public hospitals, and 1 in another type of practice.

### 3.2 Familiarity With Zygomatic Implants

Twenty‐five experts were unfamiliar with zygomatic implants and skipped the related question. As a result, consensus was based on responses from 93 experts. A strong consensus (95.7%) affirmed the necessity of specialized training (median: 7; IQR: 7–7; Figures 2 and S1).

**FIGURE 2:**
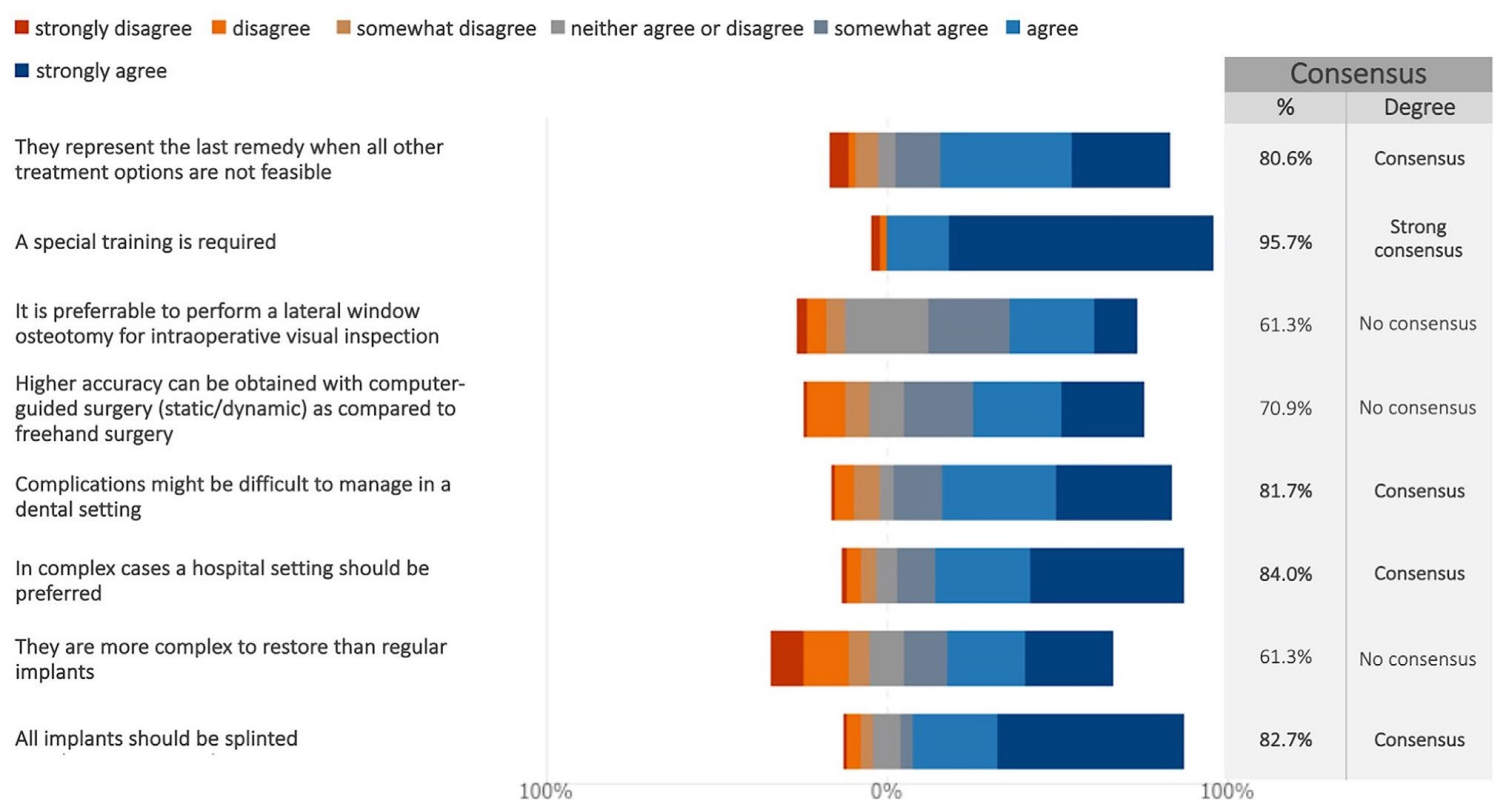
To what extent do you agree with the following statements regarding zygomatic implants?

### 3.3 Short Implants Versus Standard Implants

**FIGURE 3:**
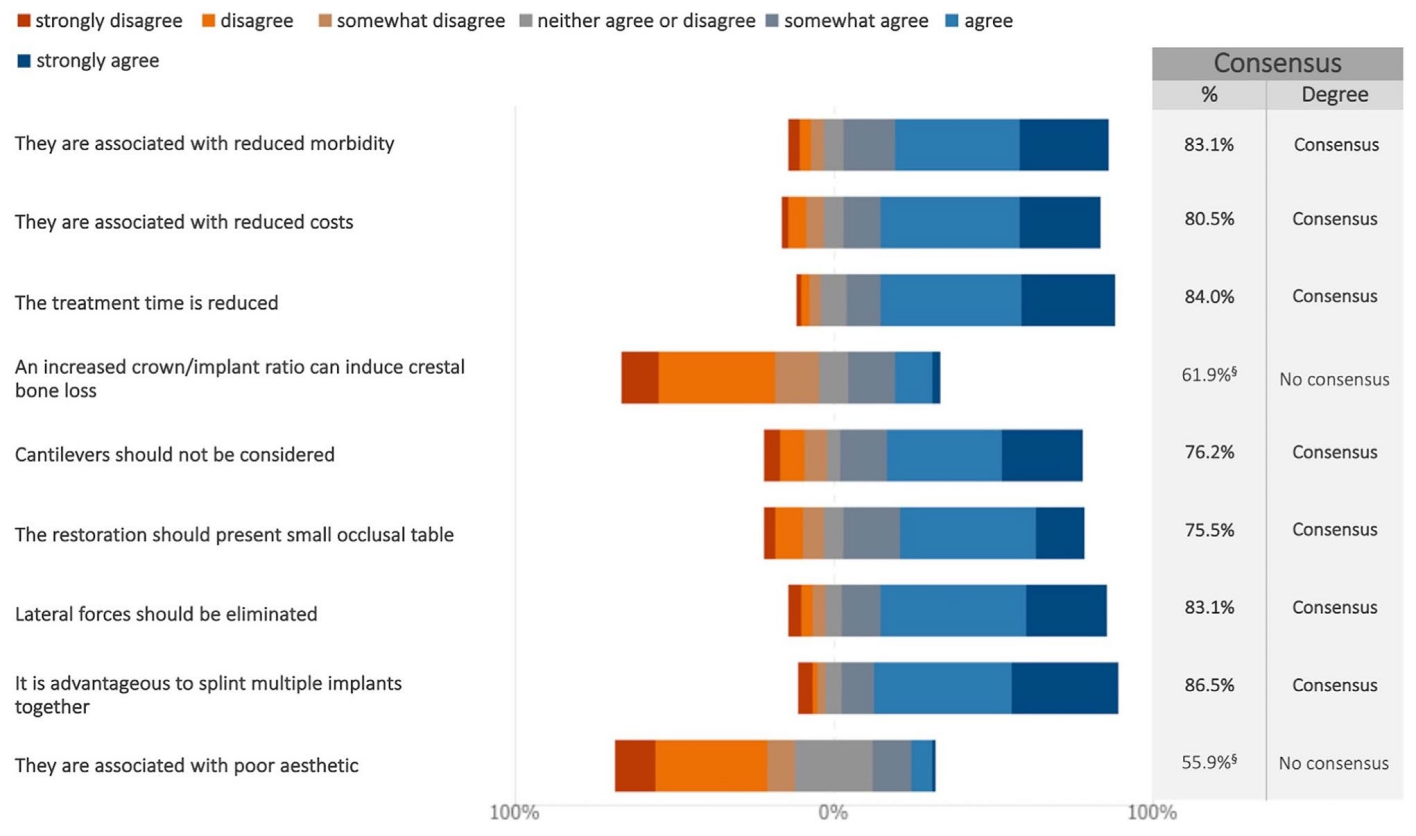
To what extent do you agree with the following statements regarding short implants as compared with standard implants and sinus lift/bone grafting? ^§^Sum of “somewhat disagree”, “disagree,” and “strongly disagree” in %.

Consensus was reached on key advantages of short implants compared to standard implants with sinus lifts or bone grafting (Figures 3 and S2). Experts agreed on benefits such as reduced mobility, lower costs, and shorter treatment times. In terms of prosthetics, consensus supported splinting multiple implants, eliminating lateral forces, avoiding cantilevers, and reducing the occlusal table.

### 3.4 Planning Phase

**FIGURE 4:**
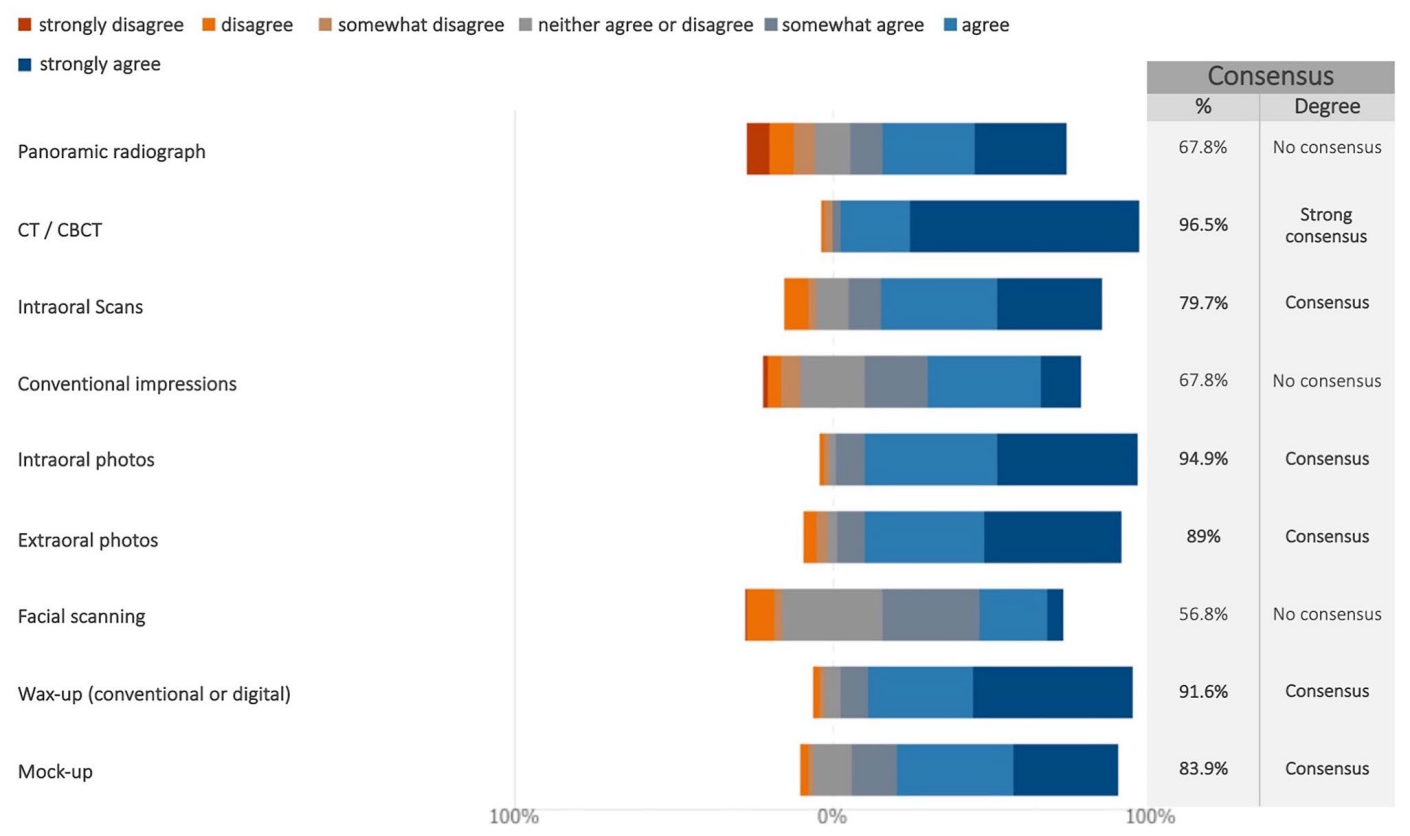
Which tool(s) should be routinely used for case study of maxillary full‐arch rehabilitation with implants?

A consensus was reached about the relevance of several tools that should be routinely used for the case study of maxillary implant‐supported full‐arch rehabilitations, comprising CT/CBCT scans (strong consensus), intraoral scans, photographs, wax‐ups, and mock‐ups (Figures 4 and S3). By contrast, no consensus was achieved for panoramic radiographs, conventional impressions, and facial scanning.

Nearly all experts (99.2%) reported assessing mouth opening and temporomandibular disorders before planning, with 89.0% doing so routinely and 10.2% in specific cases, such as when guided surgery is considered.

**FIGURE 5:**
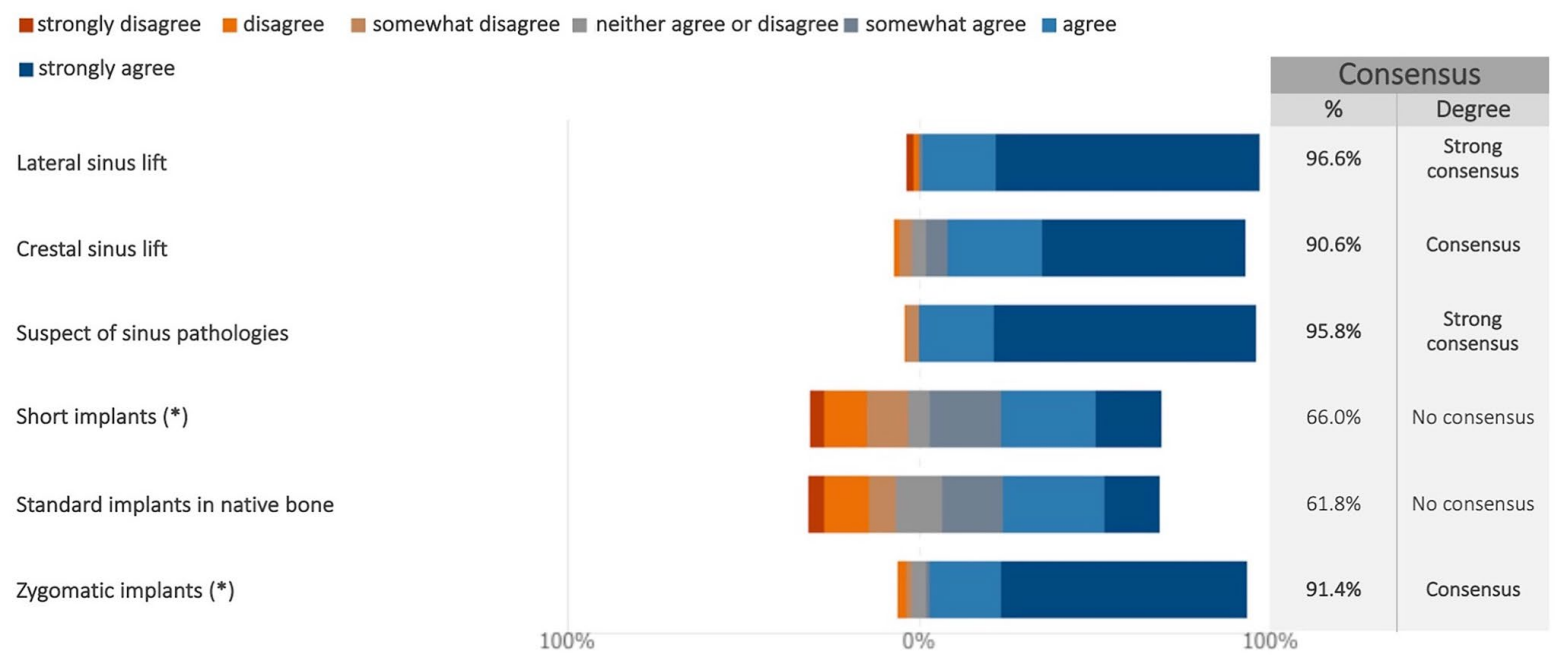
Prior to implant surgery in the posterior maxilla, in which cases do you consider necessary to check the status of the maxillary sinuses (e.g., presence of septa, ostium patency, mucous membrane thickening, and sinus pathologies)? (*) combined use of standard implants not excluded.

Consensus was reached on the necessity of preoperatively evaluating the maxillary sinuses for lateral sinus lifts and suspected pathologies (Figures 5 and S4). Consensus also supported assessment for crestal sinus lifts and zygomatic implants.

### 3.5 Guided Surgery

**FIGURE 6:**
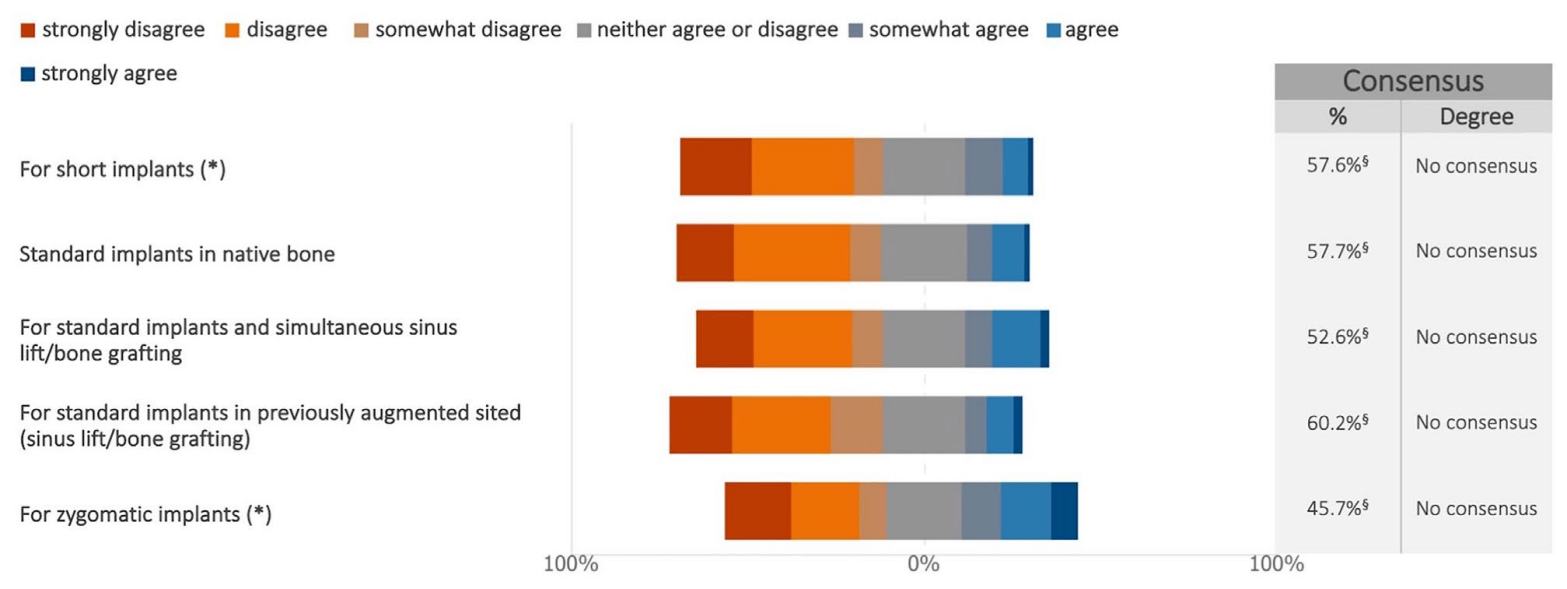
In case of multiple implant placements in fully edentulous maxilla, should freehand surgery be preferred over static/dynamic guided surgery? (*) combined use of standard implants not excluded; ^§^Sum of “somewhat disagree”, “disagree,” and “strongly disagree” in %.

No consensus was reached on whether freehand surgery should be preferred over static or dynamic guided surgery for multiple implants in a fully edentulous maxilla (Figures 6 and S5). A tendency to disagree on freehand surgery was noted.

### 3.6 Soft Tissue Augmentation

**FIGURE 7:**
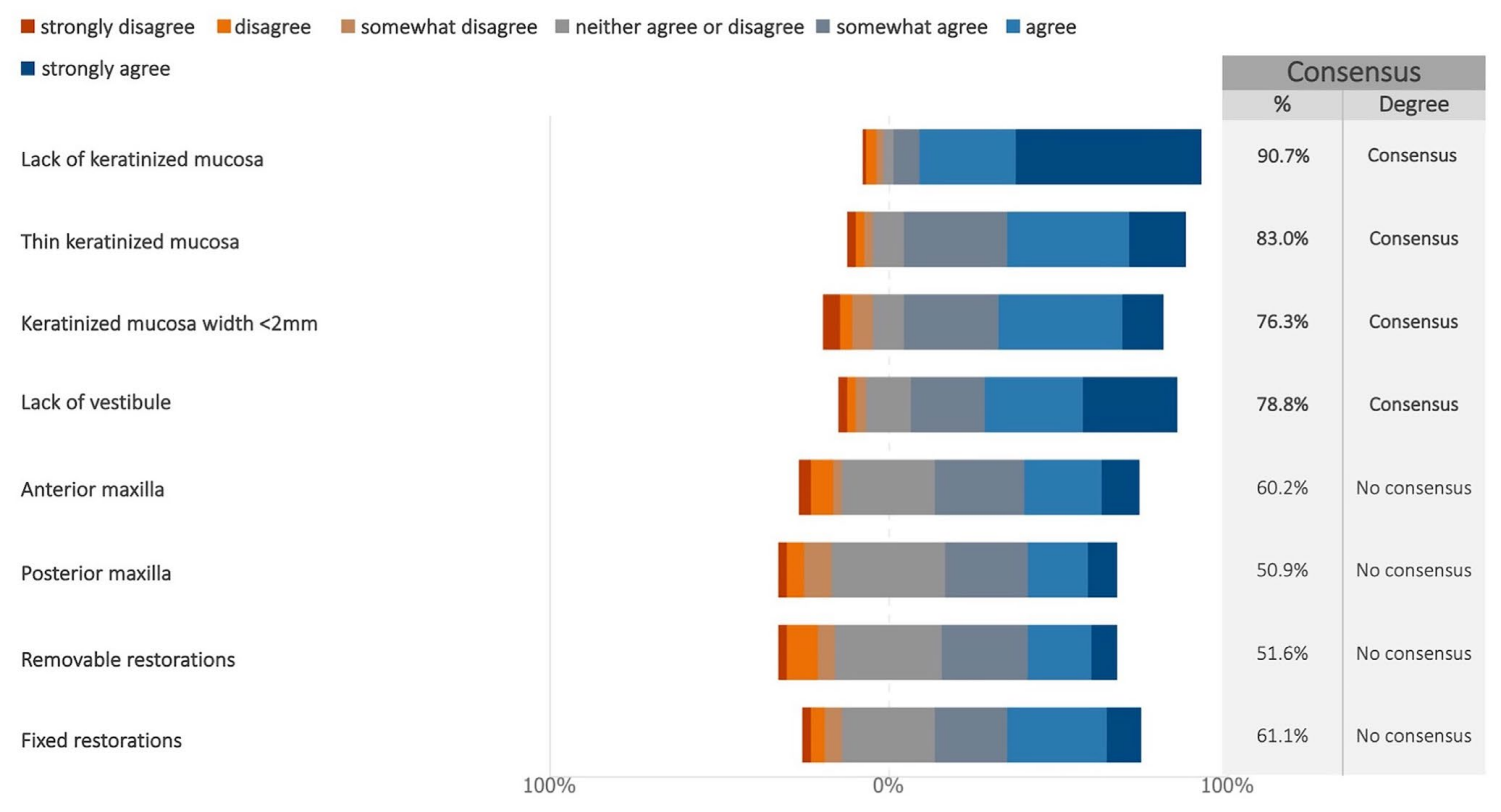
As regards soft tissue management, in which circumstances does the clinician deem soft tissue augmentation to be necessary to establish keratinized mucosa around dental implants?

Consensus supported the need for peri‐implant soft tissue augmentation in cases of absent or insufficient (< 2 mm) keratinized mucosa and lack of vestibule (Figures 7 and S6). The implant's anteroposterior location and prosthesis type were not considered determining factors.

### 3.7 Timing of Implant Placement and Loading

**FIGURE 8:**
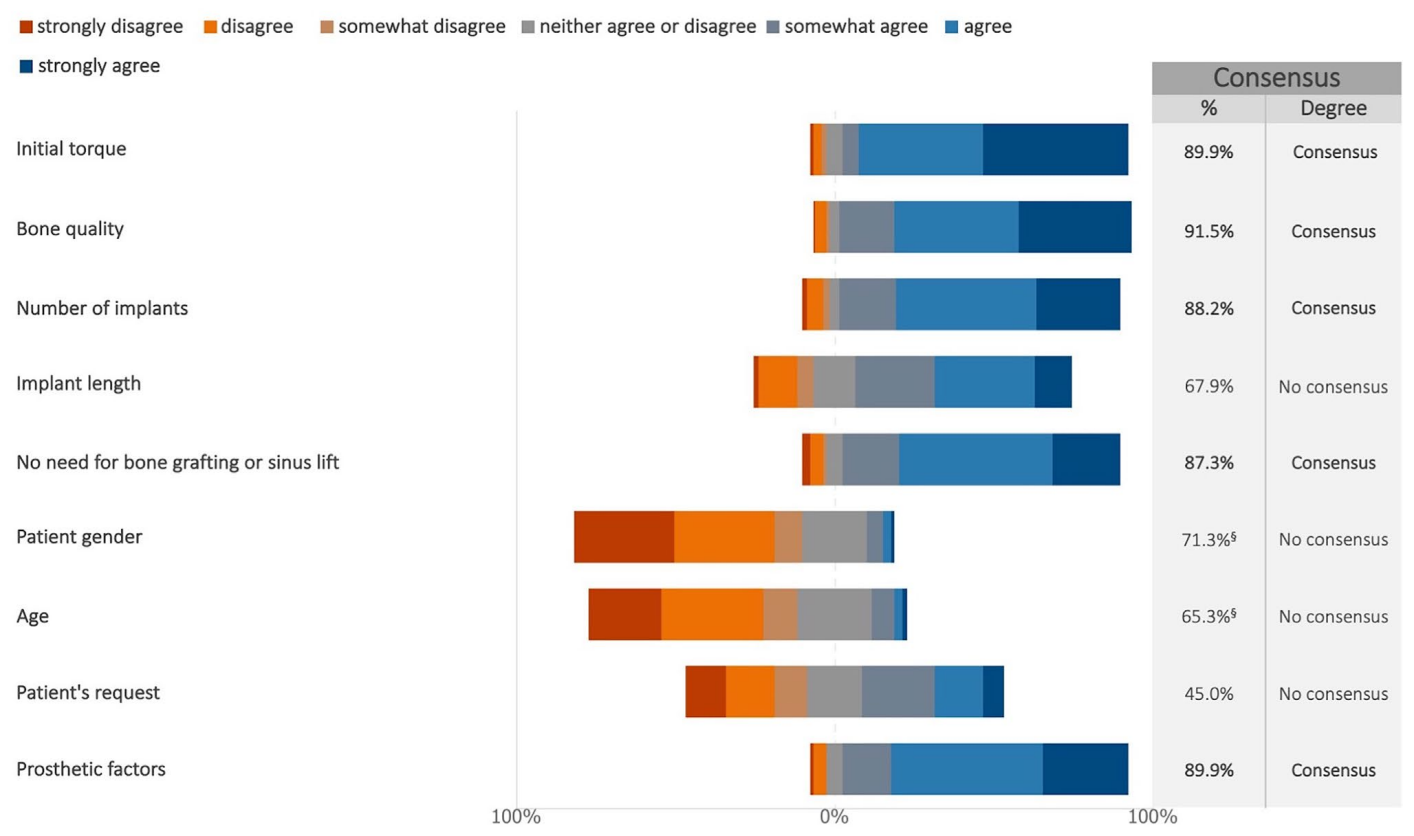
In case of multiple implant placements in fully edentulous maxilla, what are the factors that determine the decision to proceed with immediate implant loading? ^§^Sum of “somewhat disagree,” “disagree,” and “strongly disagree” in %.

A consensus was reached on several factors influencing the decision to proceed with immediate implant loading, including initial torque, bone quality, the number of implants, the absence of the need for sinus lift or other augmentation procedures, as well as prosthetic considerations (Figures 8 and S7).

**FIGURE 9:**
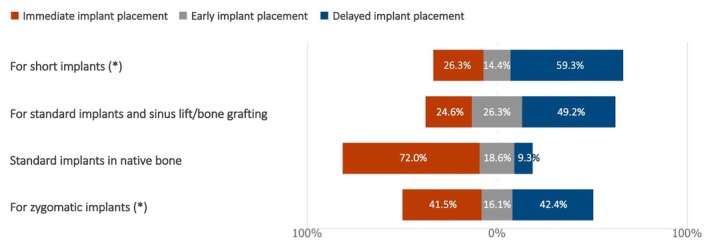
For maxillary full‐arch rehabilitation with implants, in the presence of terminal dentition in the posterior area, which protocol do you prefer among immediate, early, and delayed implant placement? (*) combined use of standard implants not excluded.

The experts were asked to indicate their preferred protocol among immediate, early, and delayed implant placement in the presence of terminal dentition in the posterior region (Figure 9). The majority of the respondents selected immediate implant placement in cases of standard implants in native bone (72.0%) and delayed implant placement for short implants (59.3%). Heterogeneous replies were recorded for zygomatic implants and augmentation procedures.

**FIGURE 10:**
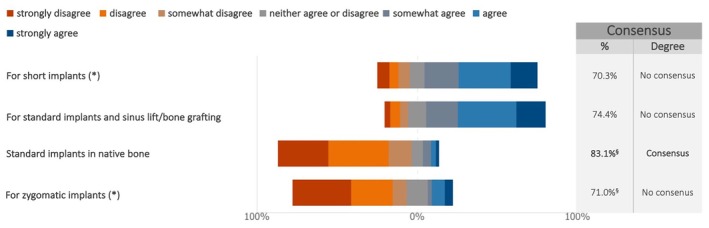
For maxillary full‐arch rehabilitation with implants, is delayed loading to be preferred over immediate loading? (*) combined use of standard implants not excluded; ^§^Sum of “somewhat disagree”, “disagree,” and “strongly disagree” in %.”

Regarding the loading protocol, consensus was reached on the restoration of standard implants placed in native bone. A similar tendency to prefer immediate loading over delayed loading was noticed for the restoration of zygomatic implants, although no consensus was achieved (Figures 10 and S8). By contrast, delayed implant loading was preferred by more than 70% of the respondents for short implants, as well as for standard implants in combination with sinus lift/bone augmentation.

### 3.8 Treatment Options

**FIGURE 11:**
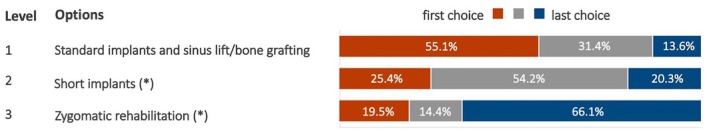
In the case of severe maxillary atrophy involving both the anterior and posterior areas, the patient's request for implant‐supported prostheses and the absence of any absolute contraindication to implant treatment, what would you generally recommend? (*) combined use of standard implants not excluded.

**FIGURE 12:**
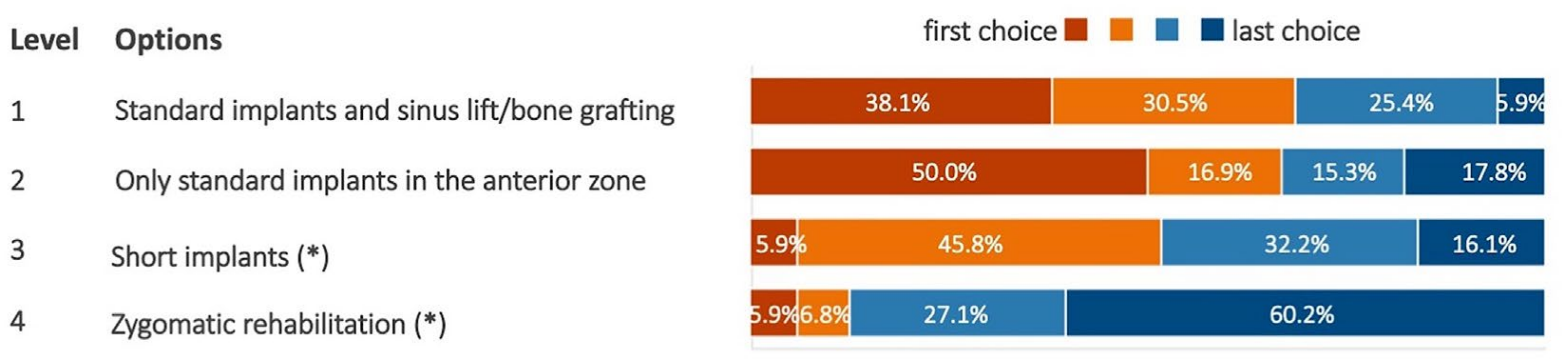
For the rehabilitation of the atrophic maxilla with a fixed implant‐supported prosthesis in the case of pneumatized maxillary sinuses and sufficient bone to position 4 implants in the anterior zone, what do you generally prefer to use? (*) combined use of standard implants not excluded.

In cases of severe maxillary atrophy, 55.1% of experts preferred standard implants with sinus lifts or bone grafting among the listed approaches (Figure 11). When sufficient anterior maxillary bone was available, 50.0% favored placing implants exclusively in this area without grafting (Figure 12). Zygomatic rehabilitations were the least preferred options in both cases.

### 3.9 Factors Influencing Selection of Treatment Procedures

**FIGURE 13:**
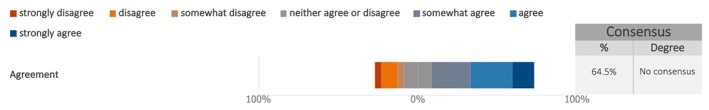
How important is the difficulty of the procedure when you choose one?

The respondents indicated that they select treatment procedures based on available evidence (31.4%) and often in combination with ease of treatment too (68.6%). However, no one supported ease of treatment alone as a justification for their selection. On the role of technical difficulty in decision‐making, 64.5% agreed that it was a factor to consider (Figures 13 and S9).

### 3.10 Antibiotic Prescription in Relation to Implant Placement

Most experts prescribed antibiotics as prophylaxis for full‐arch implant placement: 62.7% always and 32.2% in specific cases. Similarly, 62.7% routinely prescribed postoperative antibiotics, with durations ranging from 1 to 20 days (most commonly 7 days). Antibiotic use was primarily indicated for medically compromised patients and bone grafting cases.

### 3.11 Maintenance

**FIGURE 14:**
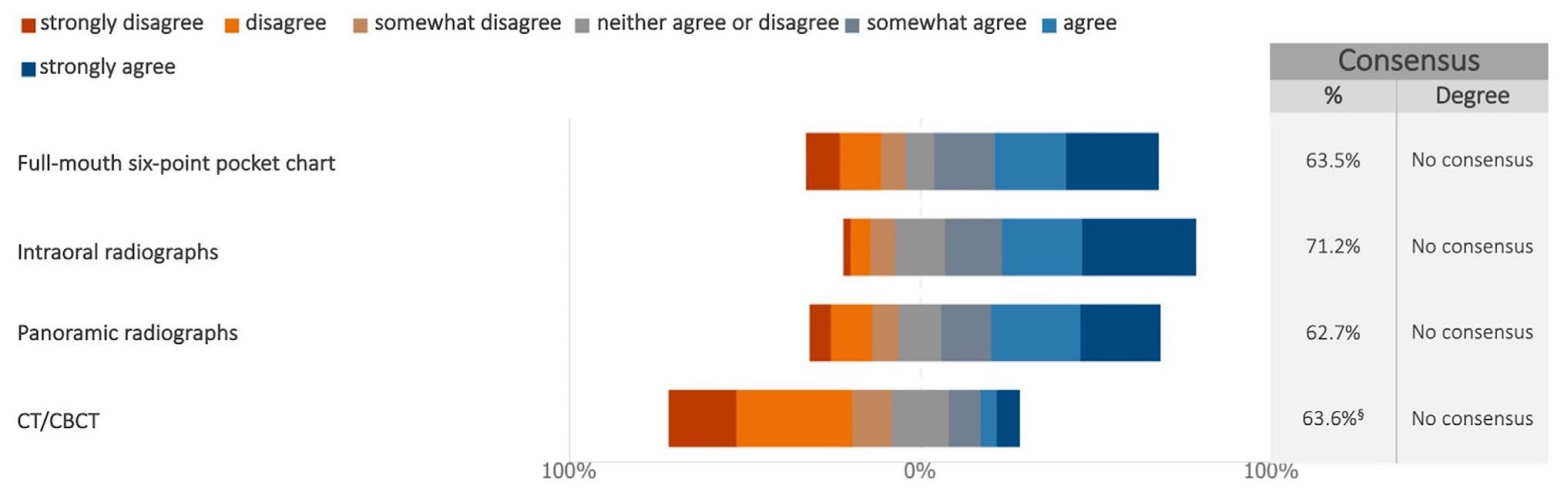
In absence of complications, are the following procedures justified at least once a year in the long‐term follow‐up? ^§^Sum of “somewhat disagree,” “disagree,” and “strongly disagree” in %.

Most experts (96.6%) offered at least annual follow‐ups after final prosthesis delivery, with 38.1% scheduling them yearly and 58.5% more frequently. No consensus was reached on the necessity of performing additional, specific clinical or radiographic exams at least once per year (Figures 14 and S10). While over half of the respondents supported intraoral and panoramic radiographs, 63.6% opposed routine CT/CBCT scans (median: 2; IQR: 2–4). Regarding professional hygiene care, 44.9% recommended twice‐yearly visits, while 32.2% advised once per year.

Expert opinions on the removal of screw‐retained prostheses for hygiene purposes varied. Overall, 51.7% recommended regular removal at intervals of 1–24 months, while 44.9% advised removal only in cases of peri‐implantitis. Specialization influenced these preferences: 62.6% of non‐prosthodontists favored regular removal, whereas 58.8% of prosthodontists recommended removal only for peri‐implantitis.

A similar pattern emerged for bar‐supported overdentures. 26.3% of experts advised regular removal for hygiene (every 1–24 months), while 66.1% recommended removal only in cases of peri‐implantitis. A small minority (7.6%) never removed the bar for hygiene. Again, non‐prosthodontists were more likely to support regular removal (37.3%), whereas prosthodontists predominantly advised removal only for peri‐implantitis (80.3%).

For both types of prostheses (i.e., screw‐retained full‐arch fixed prostheses and bar‐supported overdentures), the majority of experts asserted not to replace the prosthetic screws.

### 3.12 Home Care and Occlusal Guards

**FIGURE 15:**
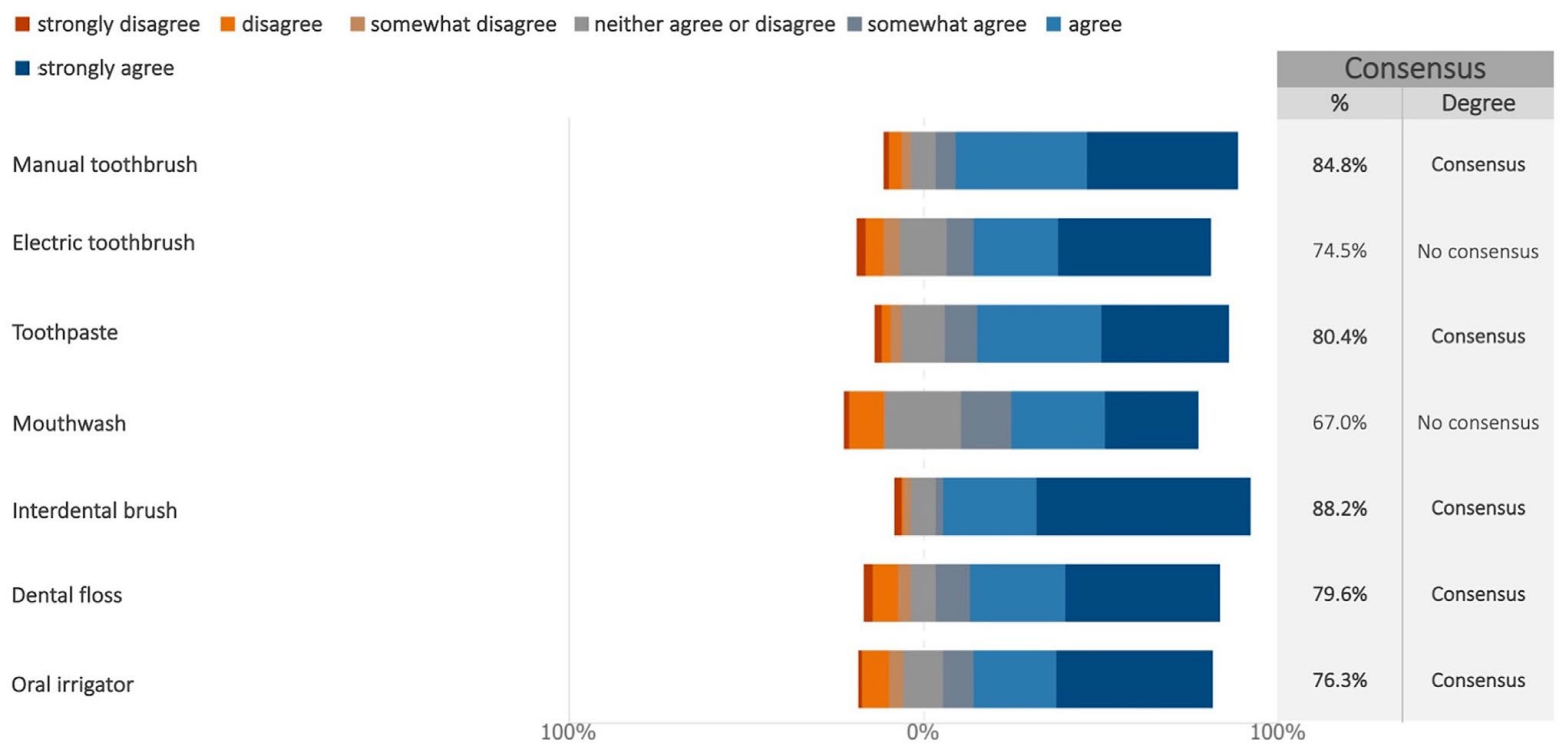
For a full arch, fixed prosthesis on implants, do you recommend the following home care devices and products to patients?

Consensus was reached on recommending manual toothbrushes, toothpaste, interdental brushes, dental floss, and oral irrigators for home care (Figures 15 and S11). However, electric toothbrushes and mouthwash did not reach consensus.

Regarding occlusal guards for maxillary implant‐supported fixed prostheses, 39.0% recommended them always, while 45.8% did so in selected cases, mainly based on the antagonist dentition or restoration material. Only 15.3% did not recommend occlusal guards.

### 3.13 Factors Affecting Patient Satisfaction

**FIGURE 16:**
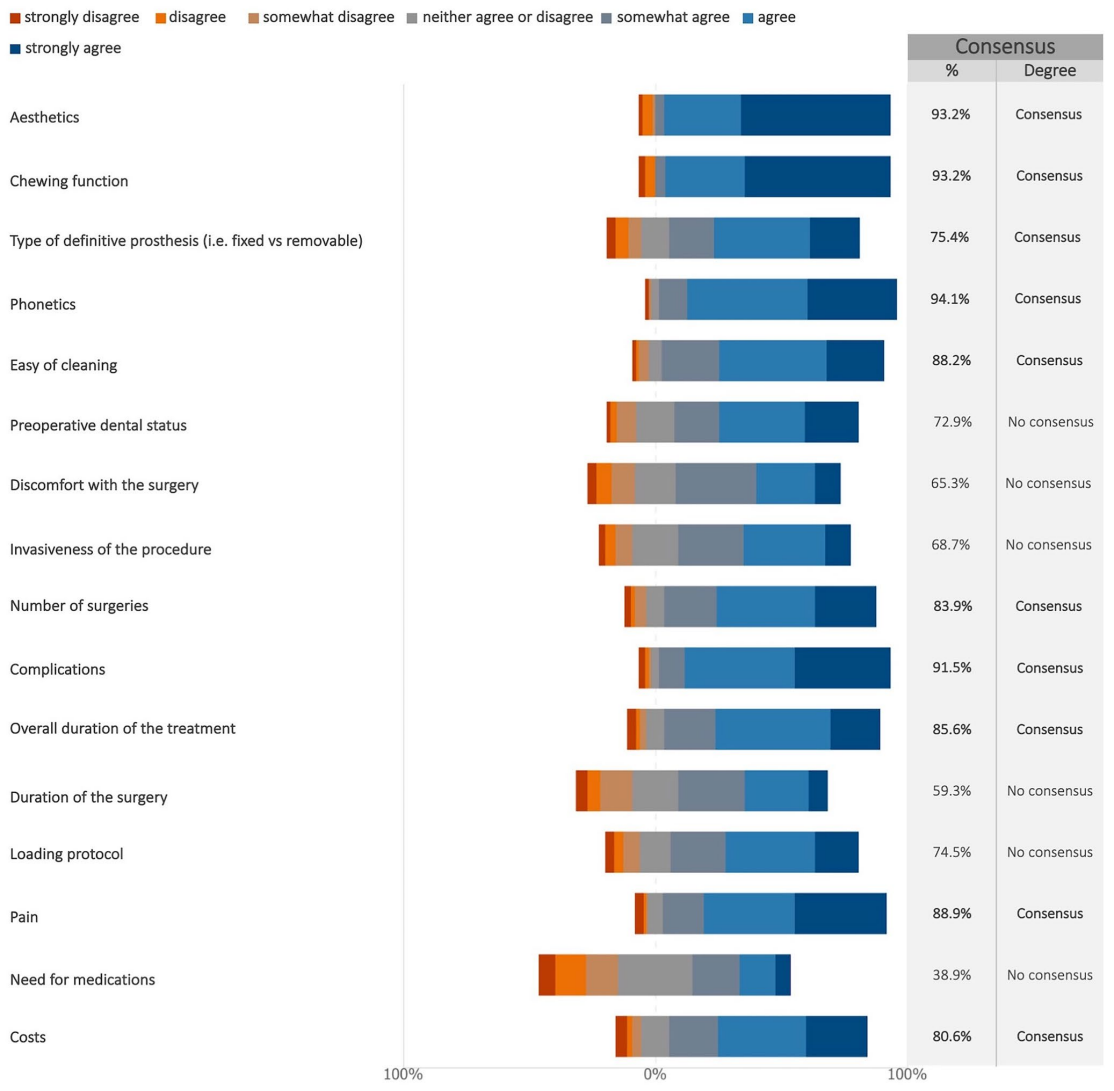
In case of maxillary full‐arch rehabilitation with dental implants, do the following factors affect the overall patient satisfaction in the short term?

Experts widely agreed that aesthetics, chewing function, phonetics, and complications significantly impact short‐term patient satisfaction (Figures 16 and S12).

### 3.14 Key Outcomes to Be Included in Future Studies

**FIGURE 17:**
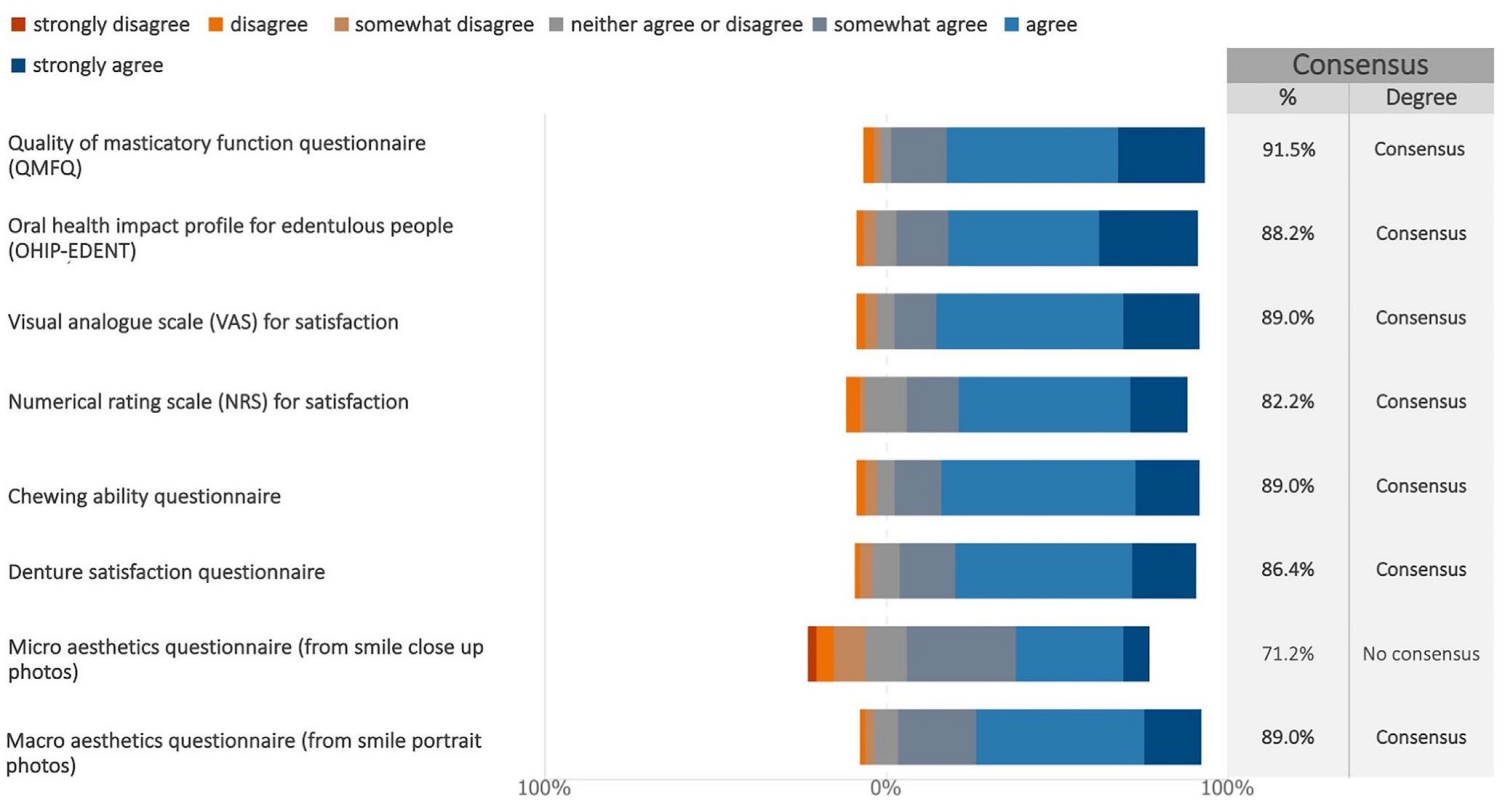
In future studies on maxillary full‐arch rehabilitation with dental implants, do you consider relevant the following PROMs?

Consensus was reached on all proposed patient‐reported outcome measures (PROMs) except the micro‐aesthetic questionnaire (Figures 17 and S13).

**FIGURE 18:**
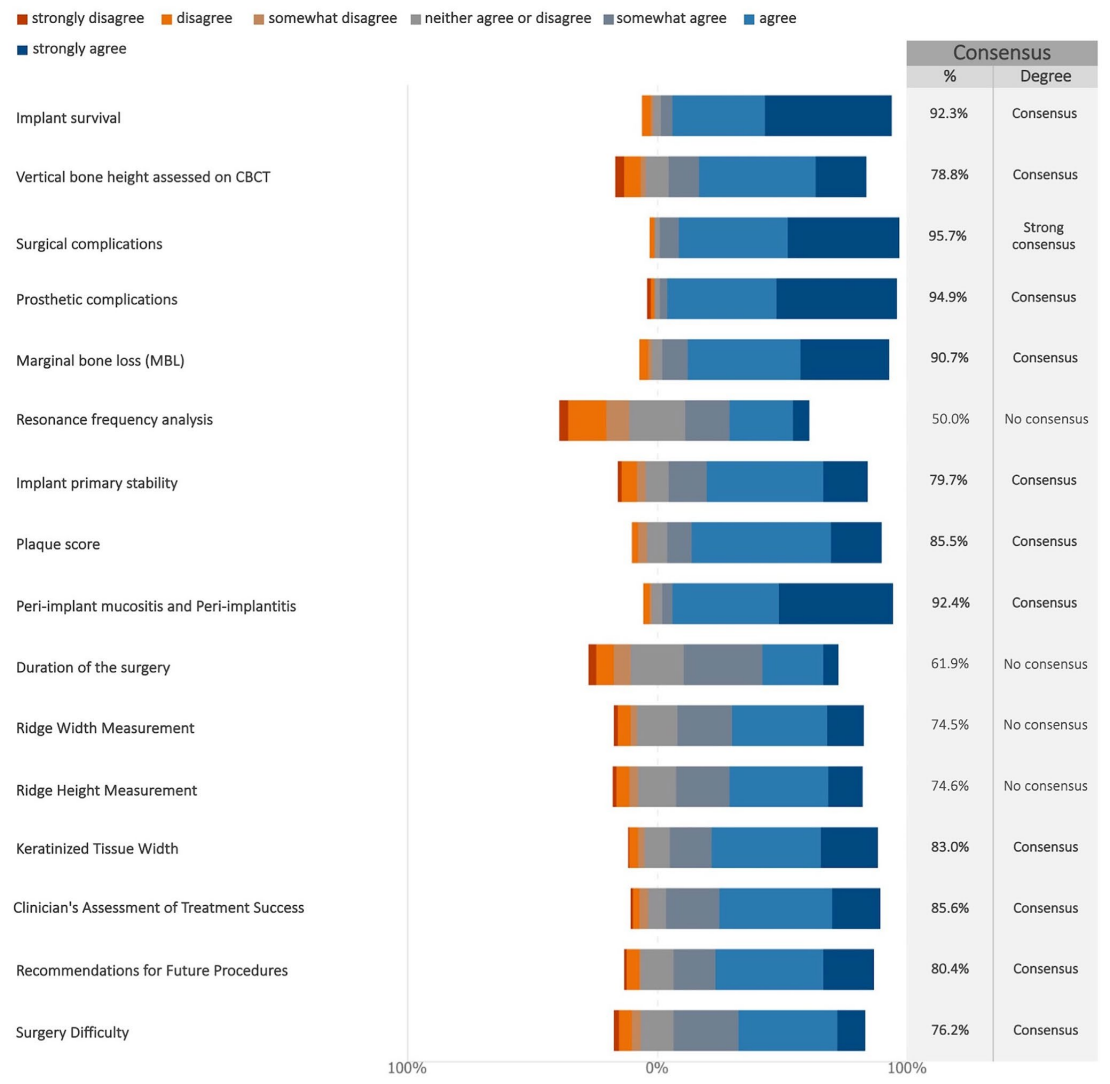
In future studies on maxillary full‐arch rehabilitation with dental implants, do you consider relevant the following clinician‐reported outcomes (ClinROs)?

For clinician‐reported outcomes (ClinROs), surgical complications reached strong consensus for inclusion. There was also consensus for inclusion of a further 12 key ClinROs as seen in Figure 18. Three of the remaining four achieved more than 60% support, whereas resonance frequency analysis only had 50% (Figures 18 and S14).

## 4 Discussion

The aim of the present study was to gather expert opinions using a single‐round survey on the use of standard, short (< 8 mm), and/or zygomatic implants supporting fixed and removable prostheses in the edentulous maxilla. The findings were then compared with existing evidence to inform a consensus development process and identify the need for further systematic reviews or research gaps. The key findings from the survey are as follows:

### 4.1 Zygomatic Implants

There was a consensus that they are the last resort solution for complex patient cases when more common treatment modalities are not feasible. In fact, strong agreement (> 95%) was reached that this treatment option requires highly specialized training and should be performed in a hospital setting. These findings are consistent with previous clinical trials (Davo et al. 2018; Fan et al. 2025), systematic reviews (Lan et al. 2021), and consensus studies (Al‐Nawas et al. 2023) on zygomatic implants. Additionally, a recent Delphi study (Testori et al. 2024) reinforced that this is not a procedure for routine practice. This may also explain why no consensus was reached on whether zygomatic implants are more complex to restore than regular implants, as most experts do not routinely restore zygomatics in their daily practice.

### 4.2 Short Implants

There was a clear consensus that short implants offer several benefits, including reduced morbidity, lower costs, and shorter treatment times. These advantages are consistent with a series of RCTs comparing short implants to longer implants in combination with sinus floor elevation (Thoma et al. 2018; Thoma, Haas, et al. 2024; Thoma et al. 2015).

From a prosthetic standpoint, the survey experts agreed that short implants should be splinted and the prostheses should have a narrow occlusal table to minimize lateral forces and avoid cantilevers. This consensus aligns with existing recommendations in the literature regarding occlusal schemes for single implants and implant‐supported fixed partial prostheses. These guidelines aim to protect the prostheses, implants, and surrounding bone from overload (Sheridan et al. 2016).

A recent RCT that combined short implants with a cantilever appears to suggest that overloading may exist, particularly in the mandible, which appears more susceptible to its negative effects (Gil et al. 2022). However, there is still no clear evidence identifying the most favorable occlusal scheme for implant‐supported prostheses (Goldstein et al. 2021), as current practice is largely based on traditional prosthodontic concepts. Notably, conventional concerns such as an increased crown/implant ratio affecting marginal bone loss seem to be losing relevance (Chen et al. 2023; Padhye et al. 2020). Despite a lack of consensus, the experts were inclined toward delayed implant placement when using short implants. This might be explained by the limited data on immediate loading with short dental implants (Cannizzaro et al. 2018).

### 4.3 Planning Phase

There was strong consensus on the routine use of CT/CBCT (> 95%) and consensus (> 80%) was reached for intraoral scans, photographs, wax‐ups, and mock‐ups for the planning phase of implant‐supported maxillary full‐arch restorations. These findings are in line with the steady and continuous evolution of computer‐assisted implant planning and placement (Jung et al. 2009; Schneider et al. 2018) enabling clinicians to plan ahead. In a previous Delphi study by the EAO on the trends in Implant Dentistry in Europe for the year 2030, there was moderate consensus (84%) that implant placement will become mainly guided (Sanz et al. 2019). A similar consensus indicating that guided surgery will be more common was also reached in a Delphi study involving professionals from Latin America (Alarcon et al. 2021). Conversely, no consensus was reached for the use of panoramic x‐rays or conventional impressions, further highlighting the establishment of digital tools in daily routine. The lack of consensus regarding facial scanning is most likely due to the currently limited data and that its added value remains to be established (Revilla‐Leon et al. 2024).

There was strong consensus among the responding experts on the importance of assessing the status of the maxillary sinuses before performing surgical interventions. This finding is largely in line with a recent Delphi study on the rehabilitation of the posterior atrophic maxilla (Testori et al. 2024). This study indicated that the sinus should be evaluated anatomically and functionally to determine the best treatment approach and avoid complications consistent with previous narrative (Testori et al. 2023) and systematic reviews (Hsu et al. 2022).

Our results emphasized the importance of assessing limited mouth opening and the presence of TMJ disorders during treatment planning. This is aligned with existing literature, as reduced mouth opening could hinder implant placement when static computer‐guided surgery or some robotic systems are employed, in particular in the posterior regions (Gargallo‐Albiol et al. 2024; Liu et al. 2024; Liu et al. 2025; Pomares‐Puig et al. 2023).

Indeed, limited mouth opening has been shown to be a significant limitation of the fully guided approach for dental implant placement and affects a considerable number of patients, especially in the posterior areas and in the older age group (Gargallo‐Albiol et al. 2024). The importance of evaluating mouth opening during the planning phase was also recognized by 85% of experts in a consensus conference focused exclusively on zygomatic implant‐supported prostheses (Aparicio and Pastorino 2023).

### 4.4 Guided Surgery

For multiple implant placements in an edentulous maxilla, there was agreement that static or dynamic‐guided surgery should be preferred over freehand placement but without reaching consensus (< 60%). This preference is consistent with a recent systematic review with meta‐analysis, which showed that static fully guided implant navigation surgery offers the highest accuracy in translating the presurgical plan to the patient, followed by static half‐guided surgery, with freehand placement being the least accurate (Gargallo‐Albiol et al. 2020). The absence of consensus (consensus threshold > 75%) may reflect the high level of experience among the clinicians surveyed. Many of these experienced professionals may feel equally confident with both guided and freehand techniques or may be reluctant to favor guided surgery over freehand placement. A previous consensus noted that static computer‐assisted surgery tends to be less accurate in fully edentulous cases compared to partially edentulous ones (Wismeijer et al. 2018). Additionally, the length and geometry of the edentulous span can influence the accuracy of digital impressions, thereby affecting the planning process (Wismeijer et al. 2018). Finally, the inherent learning curve associated with new technologies may further contribute to clinicians' reluctance to adopt guided surgery.

### 4.5 Soft Tissue Augmentation

There was consensus among the experts on the necessity of peri‐implant soft tissue augmentation to ensure a sufficient width of keratinized tissue (≥ 2 mm) and the use of vestibuloplasty to increase vestibular height. These responses were not associated with implant location (anterior or posterior) or prostheses type (fixed or removable). However, despite this expert consensus, the literature remains divided on the significance of keratinized mucosa width (KMW). Notably, the European Federation of Periodontology (EFP) S3‐level clinical practice guideline states that the effect of soft tissue augmentation (“prophylactic” soft tissue augmentations) on peri‐implant health remains unclear (Herrera et al. 2023). This uncertainty is reflected in the conflicting evidence. A recent systematic review reported that KMW less than 2 mm was associated with increased soft tissue recession, marginal bone loss, plaque accumulation, and peri‐implantitis (Ramanauskaite et al. 2022), whereas a more recent meta‐analysis suggests that the presence of keratinized mucosa (≥ 2 mm vs. < 2 mm) has minimal impact on peri‐implantitis development (Ravidà et al. 2022). Similarly, a 10‐year prospective study (Mancini et al. 2024) found that although insufficient buccal keratinized mucosa is associated with increased inflammatory parameters such as bleeding on probing (BOP), the strength of this association appears weak.

### 4.6 Timing of Implant Placement and Loading

Consensus was reached on factors affecting the feasibility of immediate loading of dental implants. These included the final insertion torque, the bone quality, the number of implants placed, and the presence or absence of augmentation procedures. The majority of the respondents preferred an immediate loading concepts for standard‐length implants placed in native bone, whereas a delayed loading was recommended whenever short implants are placed. These findings are consistent with previous systematic reviews indicating that immediate implant loading with a fixed prosthesis in the edentulous maxilla seems to be a reliable treatment alternative (Jiang et al. 2021) with similar survival rates to delayed loading (Papaspyridakos et al. 2014). A recent systematic review (Kammerer et al. 2023) found that loading protocols were linked to the surgical technique. The prevalence of immediate loading was 22.3% with the original surgical technique but increased to 89.6% when using the Anatomy‐Guided Approach (AGA) for zygomatic implant placement.

### 4.7 Treatment Options

Most experts preferred standard‐length implants in combination with sinus elevation procedures or horizontal/vertical bone grafting in case of a severely atrophic maxilla in posterior sites. Implant placement without grafting was favored in anterior sites with an expected sufficient amount of native bone. Bone augmentation techniques, such as sinus lift, and full‐arch fixed rehabilitations supported by tilted and axially placed implants in native bone have been extensively documented and are considered reliable treatment options (Aghaloo et al. 2016; Del Fabbro et al. 2022; Khoury et al. 2017; Testori et al. 2024). However, comparative studies evaluating these approaches in terms of clinical outcomes and patient‐reported outcomes are currently lacking. A recent systematic review concluded that 6‐mm implants and ≥ 10‐mm implants placed after maxillary sinus lift show similar survival and complication rates. However, it also highlighted the need for long‐term RCTs with follow‐up periods of at least 10 years to establish the long‐term efficacy of short dental implants (Ravidà et al. 2024).

Zygomatic implants and their associated rehabilitations were the least preferred treatment option. Their use has also been discussed in other consensus conferences focused on the atrophic maxilla, particularly for completely edentulous elderly or oncological patients when conventional implants are not viable (Al‐Nawas et al. 2023; Testori et al. 2024). The cumulative survival rate of zygomatic implants for rehabilitating severely atrophic maxillae has been reported to exceed 95% after more than 5 years (Chrcanovic et al. 2016; Solà Pérez et al. 2022). Patients restored with zygomatic implant‐supported full‐arch fixed prosthesis have also reported significant improvements in oral health‐related quality of life (OHRQoL) and overall satisfaction (Al‐Nawas et al. 2023; Sáez‐Alcaide et al. 2022). However, expert preference may have been influenced by other factors, such as the technical challenges involved or the potential for postoperative complications. These complications include sinusitis—reported in more than 10% of cases—soft tissue infections, paresthesia, and oroantral fistulas (Chrcanovic et al. 2016; Lan et al. 2021). The incidence rates of these complications vary across studies, and some may be underreported due to inconsistent documentation.

### 4.8 Factors Influencing Selection of Treatment Procedures

The respondents based their choice of treatment procedures on available evidence and often in combination with ease of treatment. However, ease alone was not enough to justify their choice. There was also a tendency toward agreement that technical difficulty was a factor in decision‐making but not enough to achieve consensus. Notably, few studies in implant dentistry have documented or measured procedural difficulty using visual analog scales (Cosyn et al. 2023) or numeric rating scales (Thoma, Gil, et al. 2024).

### 4.9 Antibiotic Prescription in Relation to Implant Placement

Most experts routinely prescribe antibiotics for prophylactic reasons and postoperatively, especially in medically compromised patients or when bone grafting procedures are applied.

In many countries, there are no clear or standardized guidelines regarding the prophylactic use of antibiotics in implant dentistry, leading to variability in clinical practice (Becker et al. 2024). As a result, many practitioners continue to administer prophylactic antibiotics routinely, particularly in medically compromised patients or those undergoing complex procedures such as bone grafting (Becker et al. 2024).

### 4.10 Maintenance

Most experts recommended annual or more frequent regular examinations after the insertion of the final implant‐supported fixed prosthesis. This is in line with the European Federation of Periodontology (EFP) S3 guidelines, which emphasize the importance of supportive peri‐implant care to minimize the risk of developing peri‐implant diseases in patients with healthy peri‐implant tissues (Herrera et al. 2023). However, no consensus (63.5%) was reached regarding the necessity of performing a full‐mouth pocket chart at least once a year during follow‐up. This lack of agreement may reflect the individualized nature of supportive peri‐implant care, which should be tailored to patient‐, implant‐, and restoration‐specific risk profiles and needs, with recommended recall intervals varying between 3, 6, or 12 months (Herrera et al. 2023).

The opinion varied among experts on whether annual professional hygiene procedures and radiographic examinations are justified during long‐term follow‐up in the absence of complications. There was a general tendency, however, to discourage the routine use of CT/CBCT during the follow‐up in the absence of complications, which is aligned with recommendations from several scientific dental societies (Ahmad and Chapokas 2016; Bornstein et al. 2014; Tyndall et al. 2012). There are further recommendations that the use of CT/CBCT following dental implant placement should be limited to specific postoperative complications, with dose‐reduction strategies implemented to minimize unnecessary radiation exposure while maintaining diagnostic accuracy (Bornstein et al. 2017).

Opinions differed on the necessity of regularly removing screw‐retained fixed prostheses and bar‐supported overdentures for professional maintenance. Prosthodontists are generally less inclined than non‐prosthodontists to advocate for routine prosthesis removal. This difference is likely due to variations in training, as periodontists are often more critical regarding peri‐implant health. However, in cases of major biological complications (e.g., peri‐implantitis), the removal of prostheses and bar removal is considered essential (Bidra et al. 2016; Piermatti et al. 2016).

### 4.11 Home Care and Occlusal Guards

Consensus was reached on recommending manual toothbrushes, interdental brushes, dental floss, and oral irrigators, while no consensus was reached on the use of electric toothbrushes and mouthwash. Data on toothbrushes are particularly noteworthy. Although much of the existing literature focuses on gingivitis rather than mucositis reduction, electric toothbrushes, particularly those with oscillating‐rotating systems, generally show better performance compared to manual toothbrushes (Grender et al. 2020; Thomassen et al. 2022; Zou et al. 2024). This advantage is especially evident in certain populations, such as elderly individuals and those with intellectual disabilities (Yeh et al. 2024).

Currently, traditional manual methods for removing interproximal plaque, such as dental floss and interdental brushes, remain the clinical standard for maintaining peri‐implant health (Perussolo and Donos 2024). However, it is essential to consider, from the planning phase onward, especially in cases of full‐arch or partial fixed prostheses with pontics, how to ensure proper access for hygiene instruments (Herrera et al. 2023; Soares et al. 2024).

Oral irrigators used in combination with mechanical tooth brushing have been demonstrated to effectively manage peri‐implant mucositis by improving plaque control and reducing inflammation and bleeding on probing, compared to brushing alone (Gandhi et al. 2025; Gennai et al. 2023). The benefits of oral irrigators with water or additional antimicrobials seem particularly beneficial during the maintenance phase of daily at‐home care (Araújo et al. 2024; Soares et al. 2024).

Most experts based their recommendation of occlusal guards on the composition of the dentitions and restorative materials in the opposing jaw. While recommendations exist for occlusal guards for bruxing dentate patients to minimize unfavorable loads and occlusal wear of restorative materials (Berzaghi et al. 2025; Sutthiboonyapan and Wang 2019), there is a significant lack of high‐quality evidence to support the same routine use for bruxers who have undergone dental implant rehabilitation (Häggman‐Henrikson et al. 2024; Mesko et al. 2014; Sutthiboonyapan and Wang 2019).

### 4.12 Factors Affecting Patient Satisfaction

Consensus (90%) was reached on the key factors influencing short‐term patient satisfaction, including aesthetics, chewing function, phonetics, and complications. Recently, there has been a growing focus in medicine on capturing subjective patient experiences through patient‐reported outcomes (PROs) using PROMs—standardized questionnaires designed to assess patient perspectives (Weinfurt and Reeve 2022). These measures provide deeper insights into treatment challenges and aesthetic outcomes in implant therapy (Cosyn et al. 2023; Thoma, Gil, et al. 2024). This trend aligns with previous systematic reviews highlighting the increasing interest in PROMs within implant dentistry (De Bruyn et al. 2015; Feine et al. 2018; Yao et al. 2018). It has been suggested that collecting and sharing PROM data with clinicians can enhance patient‐clinician communication, ultimately improving treatment processes and outcomes (Nelson et al. 2015; Sutherland and Till 1993). For example, PROMs can help patients identify what matters most to them and express their concerns more effectively than traditional outcome measures, empowering them to engage more actively in their treatment discussions (Feldman‐Stewart and Brundage 2009; Santana and Feeny 2014).

### 4.13 Fundamental Outcomes for Future Studies

It was positive to find that there was consensus among the responding experts for inclusion of most of the PROMs listed. This lends support for a realization among dental professionals of the importance of a patient‐centered approach to implant therapy. The consensus for inclusion of different PROs such as aesthetics, chewing ability, and denture satisfaction further highlights the importance of consideration of the patients' perspective on treatment outcomes.

There was also broad consensus among the experts for inclusion of 12 of the proposed ClinROs. With 68.6% of the respondents working in private clinics, this suggests that inclusion of these outcomes is deemed important and relevant to clinical practice as well as to university and hospital settings. These outcomes were also aligned with the implant dentistry core outcome set and measurement identified in the ID‐COSM initiative (Tonetti et al. 2023).

### 4.14 Limitations

This study has several limitations. Although experts from different countries were involved, the sample is not representative (Akins et al. 2005). Geographic variations in clinical practice were not analyzed, as the survey was anonymous and no geographic data were collected. In many cases, only one expert per country was invited, and requesting information on geographic origin could have compromised anonymity. Additionally, the presence of participants with dual specialization may have diluted the clarity of discipline‐specific perspectives; therefore, subgroup comparisons were not conducted.

This survey employed a single round to prioritize brevity and efficiency; however, this approach may limit the depth of consensus usually achieved through multiple iterative rounds, where participants can provide feedback, refine their opinions, and introduce new ideas. Although this is a recognized limitation, these topics were further discussed in person during the consensus meeting. Another limitation was that 25% of experts lacked experience with zygomatic implants, potentially introducing bias. To minimize this, these participants were excluded from responding to the item specifically addressing zygomatic implants (item 5).

Finally, the response rate of 59.3%, while higher than in similar studies in implant dentistry (e.g., Sanz et al. 2019, with 47.5% and 44.1% response rates in two rounds), may still limit the generalizability of the findings and introduce response bias. A possible factor for the relatively modest response rate could be the order in which the questionnaire items were presented (McColl et al. 2001). Simply put, questions placed at the beginning of a survey may influence a respondent's motivation to complete it, either positively or negatively (Brookes et al. 2018). To minimize such bias, initial piloting and randomization of survey items are recommended. These considerations highlight the need for cautious interpretation of the current findings and suggest that future studies may benefit from employing strategies to improve expert participation and adopting multi‐round designs to strengthen consensus outcomes.

## 5 Conclusions

This survey evaluated the responses from selected experts regarding the treatment approaches used for the rehabilitation of the edentulous maxilla using standard, short, and/or zygomatic implants, providing insights for future consensus and guideline development. The main practical findings were:

Zygomatic implants were regarded as a last‐resort option, requiring specialized training and hospital‐based care. Short implants were favored for reduced morbidity, lower cost, and shorter treatment times, with consensus on splinting, minimizing cantilevers, and limiting lateral forces. CT/CBCT imaging, intraoral scanning, and comprehensive preoperative assessments, including sinus and TMJ evaluations, were strongly supported. Although guided surgery was generally preferred, no consensus was reached, reflecting variability in experience and technological limitations for fully edentulous cases. Soft tissue augmentation to achieve ≥ 2 mm keratinized mucosa was widely supported.

Immediate loading was favored for standard implants in native bone, while delayed loading was preferred for short implants and grafted sites. Standard implants with sinus lift or grafting were preferred for severe atrophy, while zygomatic implants were less favored due to technical demands and potential complications. Perioperative antibiotic use was common, especially in medically compromised patients. Annual or more frequent maintenance was recommended, though consensus was lacking on full‐mouth probing, radiographs, and prosthesis removal. Routine CT/CBCT during maintenance was generally discouraged.

Consensus was achieved on the recommended use of manual hygiene aids while a consensus for recommending electric toothbrushes and mouth rinses was not reached. Occlusal guard prescriptions were individualized. Aesthetics, function, phonetics, and complications were identified as primary determinants of patient satisfaction. The need for reporting of both clinician and patient‐reported outcomes in future research reached consensus in the expert survey, reinforcing the importance of patient‐centered care.

These findings reflect current expert perspectives and identify priorities for future research and clinical guideline development.

## Author Contributions

**Franz J. Strauss:** conceptualization, investigation, methodology, formal analysis, data curation, writing – review and editing, writing – original draft, supervision. **Giulia Brunello:** conceptualization, methodology, visualization, writing – review and editing, software, data curation, supervision, writing – original draft. **Daniel S. Thoma:** supervision, validation, writing – review and editing. **Ina Kopp:** writing – review and editing, methodology, supervision. **Charlotte Stilwell:** supervision, validation, writing – review and editing. **Ronald E. Jung:** supervision, resources, project administration, funding acquisition, conceptualization. **Hom‐Lay Wang:** resources, data curation, conceptualization, writing – review and editing, project administration, funding acquisition. **Frank Schwarz:** project administration, data curation, resources, conceptualization, writing – review and editing, validation, funding acquisition.

## Ethics Statement

The protocol was submitted to and approved by the Ethics Committee of the University of Düsseldorf (Protocol no. 2024‐2973).

## Conflicts of Interest

The authors declare no conflicts of interest.

## Supporting information


**Appendix S1:** clr70015‐sup‐0001‐Supinfo01.pdf.

**Appendix S2:** clr70015‐sup‐0002‐Supinfo02.docx.

## Data Availability

The data that support the findings of this study are available from the corresponding author upon reasonable request.
