# Supplementary material for: 1st Global Consensus for Clinical Guidelines for the Rehabilitation of the Edentulous Maxilla: A Single‐Round Survey on Standard, Short, and Zygomatic Implant‐Supported Prostheses
Source: Clin Oral Implants Res. 2026 Feb 24;37(Suppl 30):S135–54. doi: 10.1111/clr.70015 (PMC12930133; doi:10.1111/clr.70015)
Supplement: Supplementary file 1 — Appendix S1: clr70015‐sup‐0001‐Supinfo01.pdf. [file CLR-37-S135-s002.pdf]

Survey 2 – 1st Global Consensus for Clinical Guidelines 2025

Short vs standard and zygomatic implants

* Required

The present questionnaire aims at investigating the opinion of expert clinicians regarding different treatment modalities for maxillary full-arch rehabilitation with implants. In particular **we aim at exploring your opinion on rehabilitations supported by zygomatic implants and short implants (<8 mm).** Please be aware that when we refer to these solutions, the combined use of standard implants is not excluded and indicated with (*).

1. If you click on "yes", it means that you consent to participate in the survey, otherwise it ends here.

   *

   ◯ Yes

2. Professional specialisation *

   Multiple choice possible

   ☐ Periodontology

   ☐ Prosthodontics

   ☐ Oral surgery

   ☐ Oral and maxillofacial surgery

   ☐ None (general practitioner)

   ☐ Other

3. Please specify your professional working environment. *

   Multiple choice possible

   ☐ University

   ☐ Public hospital

   ☐ Private clinic

   ☐ Other

4. Are you familiar with zygomatic implants? *

   ◯ Yes

   ◯ No, I prefer to skip the next question about zygomatic implants

5. To what extent do you agree with the following statements regarding zygomatic implants? *

Per each answer, please indicate your level of agreement with a score from "strongly disagree" to "strongly agree" ***Make sure to slide the table all the way to the right to see all score levels, including "strongly agree."***

	strongly disagree	disagree	somewhat disagree	neither agree or disagree	somewhat agree	agree	strongly agree
They represent the last remedy when all other treatment options are not feasible	◯	◯	◯	◯	◯	◯	◯
A special training is required	◯	◯	◯	◯	◯	◯	◯
It is preferrable to perform a lateral window osteotomy for intraoperative visual inspection	◯	◯	◯	◯	◯	◯	◯
Higher accuracy can be obtained with computer-guided surgery (static/dynamic) as compared to freehand surgery	◯	◯	◯	◯	◯	◯	◯
Complications might be difficult to manage in a dental setting	◯	◯	◯	◯	◯	◯	◯
In complex cases a hospital setting should be preferred	◯	◯	◯	◯	◯	◯	◯
They are more complex to restore than regular implants	◯	◯	◯	◯	◯	◯	◯
All implants should be splinted	◯	◯	◯	◯	◯	◯	◯

6. To what extent do you agree with the following statements regarding short implants as compared with standard implants and sinus lift/bone grafting? *

Per each answer, please indicate your level of agreement with a score from "strongly disagree" to "strongly agree"

	strongly disagree	disagree	somewhat disagree	neither agree or disagree	somewhat agree	agree	strongly agree
They are associated with reduced morbidity	◯	◯	◯	◯	◯	◯	◯
They are associated with reduced costs	◯	◯	◯	◯	◯	◯	◯
The treatment time is reduced	◯	◯	◯	◯	◯	◯	◯
An increased crown/implant ratio can induce crestal bone loss	◯	◯	◯	◯	◯	◯	◯
Cantilevers should not be considered	◯	◯	◯	◯	◯	◯	◯
The restoration should present small occlusal table	◯	◯	◯	◯	◯	◯	◯
Lateral forces should be eliminated	◯	◯	◯	◯	◯	◯	◯
It is advantageous to splint multiple implants together	◯	◯	◯	◯	◯	◯	◯
They are associated with poor aesthetic	◯	◯	◯	◯	◯	◯	◯

7. Which tool(s) should be routinely used for case study of maxillary full-arch rehabilitation with implants? *

Per each answer, please indicate your level of agreement with a score from "strongly disagree" to "strongly agree" ***Make sure to slide the table all the way to the right to see all score levels, including "strongly agree."***

	strongly disagree	disagree	somewhat disagree	neither agree or disagree	somewhat agree	agree	strongly agree
Panoramic radiograph	◯	◯	◯	◯	◯	◯	◯
CT / CBCT	◯	◯	◯	◯	◯	◯	◯
Intraoral Scans	◯	◯	◯	◯	◯	◯	◯
Conventional impressions	◯	◯	◯	◯	◯	◯	◯
Intraoral photos	◯	◯	◯	◯	◯	◯	◯
Extraoral photos	◯	◯	◯	◯	◯	◯	◯
Facial scanning	◯	◯	◯	◯	◯	◯	◯
Wax-up (conventional or digital)	◯	◯	◯	◯	◯	◯	◯
Mock-up	◯	◯	◯	◯	◯	◯	◯

8. Do you check the mouth opening and pre-existing TMJ disorders prior to the planning? *

- ◯ Yes, always
- ◯ Yes, in selected cases
- ◯ When guided surgery is considered
- ◯ No, never

9. If "yes, in selected cases", which ones? *

10. Prior to implant surgery in the posterior maxilla, in which cases do you consider necessary to check the status of the maxillary sinuses (e.g. presence of septa, ostium patency, mucous membrane thickening, sinus pathologies)? *

Per each answer, please indicate your level of agreement with a score from "strongly disagree" to "strongly agree" ***Make sure to slide the table all the way to the right to see all score levels, including "strongly agree."***

	strongly disagree	disagree	somewhat disagree	neither agree or disagree	somewhat agree	agree	strongly agree
Lateral sinus lift	◯	◯	◯	◯	◯	◯	◯
Crestal sinus lift	◯	◯	◯	◯	◯	◯	◯
Suspect of sinus pathologies	◯	◯	◯	◯	◯	◯	◯
Short implants (*)	◯	◯	◯	◯	◯	◯	◯
Standard implants in native bone	◯	◯	◯	◯	◯	◯	◯
Zygomatic implants (*)	◯	◯	◯	◯	◯	◯	◯

11. In case of multiple implant placements in fully edentulous maxilla, should freehand surgery be preferred over static/dynamic guided surgery? *

Per each answer, please indicate your level of agreement with a score from "strongly disagree" to "strongly agree"

	strongly disagree	disagree	somewhat disagree	neither agree or disagree	somewhat agree	agree	strongly agree
For short implants (*)	◯	◯	◯	◯	◯	◯	◯
Standard implants in native bone	◯	◯	◯	◯	◯	◯	◯
For standard implants and simultaneous sinus lift/bone grafting	◯	◯	◯	◯	◯	◯	◯
For standard implants in previously augmented sited (sinus lift/bone grafting)	◯	◯	◯	◯	◯	◯	◯
For zygomatic implants (*)	◯	◯	◯	◯	◯	◯	◯

12. In case of multiple implant placements in fully edentulous maxilla, which are the factors that determine the decision to proceed with immediate implant loading? *

Per each answer, please indicate your level of agreement with a score from "strongly disagree" to "strongly agree"
Make sure to slide the table all the way to the right to see all score levels, including "strongly agree."

	strongly disagree	disagree	somewhat disagree	neither agree or disagree	somewhat agree	agree	strongly agree
Initial torque	◯	◯	◯	◯	◯	◯	◯
Bone quality	◯	◯	◯	◯	◯	◯	◯
Number of implants	◯	◯	◯	◯	◯	◯	◯
Implant length	◯	◯	◯	◯	◯	◯	◯
No need for bone grafting or sinus lift	◯	◯	◯	◯	◯	◯	◯
Patient gender	◯	◯	◯	◯	◯	◯	◯
Age	◯	◯	◯	◯	◯	◯	◯
Patient's request	◯	◯	◯	◯	◯	◯	◯
Prosthetic factors	◯	◯	◯	◯	◯	◯	◯

13. In case of severe maxillary atrophy involving both the anterior and posterior areas, patient's request for implant-supported prostheses and absence of any absolute contraindication to implant treatment, what would you generally recommend?
Please order the three options based on your preference - best one as first *

- Short implants (*)
- Standard implants and sinus lift/bone grafting
- Zygomatic rehabilitation (*)

14. For the rehabilitation of the atrophic maxilla with fixed implant-supported prosthesis in case of pneumatized maxillary sinuses and sufficient bone to position 4 implants in the anterior zone, what do you generally prefer to use?
Please order the four options based on your preference - best one as first. *

- Only standard implants in the anterior zone
- Short implants (*)
- Standard implants and sinus lift/bone grafting
- Zygomatic rehabilitation (*)

15. Do you routinely prescribe antibiotics as prophylaxis during multiple implant placement for the rehabilitation of the fully edentulous maxilla? *

◯ Always

◯ Yes, in the following circumstances listed below

◯ Never

16. Please indicate which circumstances *

Multiple selections possible

- [ ] Medically compromised patients (e.g. risk of infective endocarditis, immunocompromised, diabetes, etc.)
- [ ] Zygomatic implants
- [ ] Bone grafting
- [ ] Sinus lift independently of the presence of infection
- [ ] Sinus lift in presence of sinus infection
- [ ] Immediate implant placement
- [ ] Immediate implant placement only if involving infected sockets
- [ ] Other

17. Do you routinely prescribe postoperative antibiotics after multiple implant placement for the rehabilitation of the fully edentulous maxilla? *

- ◯ Always
- ◯ Yes, in the following circumstances listed below...
- ◯ Never

18. Please indicate which circumstances *

Multiple selections possible

- [ ] Medically compromised patients (e.g. risk of infective endocarditis, immunocompromised, diabetes, etc.)
- [ ] Zygomatic implants
- [ ] Bone grafting
- [ ] Sinus lift independently of the presence of infection
- [ ] Sinus lift in presence of sinus infection
- [ ] Immediate implant placement
- [ ] Immediate implant placement only if involving infected sockets
- [ ] Other

19. If "yes", please specify for how many days. *

The value must be a number

20. For maxillary full-arch rehabilitation with implants, in presence of terminal dentition in the posterior area which protocol do you prefer among immediate, early and delayed implant placement? *

Per each question, please select your preferred option

	Immediate implant placement	Early implant placement	Delayed implant placement
Short implants (*)	◯	◯	◯
For standard implants and sinus lift/bone grafting	◯	◯	◯
Standard implants in native bone	◯	◯	◯
For zygomatic implants (*)	◯	◯	◯

21. For maxillary full-arch rehabilitation with implants, is delayed loading to be preferred over immediate loading? *

Per each answer, please indicate your level of agreement with a score from "strongly disagree" to "strongly agree" ***Make sure to slide the table all the way to the right to see all score levels, including "strongly agree."***

	strongly disagree	disagree	somewhat disagree	neither agree or disagree	somewhat agree	agree	strongly agree
For short implants (*)	◯	◯	◯	◯	◯	◯	◯
For standard implants and sinus lift/bone grafting	◯	◯	◯	◯	◯	◯	◯
For standard implants in native bone	◯	◯	◯	◯	◯	◯	◯
For zygomatic implants (*)	◯	◯	◯	◯	◯	◯	◯

22. After the delivery of the final restoration, how often your implant patients are generally offered follow-up? *

◯ More than once a year

◯ Once a year

◯ Every other year or more rarely

◯ In case of suspect of complications

23. In absence of complications, are the following procedures justified at least once a year in the long-term follow-up? *

Per each answer, please indicate your level of agreement with a score from "strongly disagree" to "strongly agree" ***Make sure to slide the table all the way to the right to see all score levels, including "strongly agree."***

	strongly disagree	disagree	somewhat disagree	neither agree or disagree	somewhat agree	agree	strongly agree
Full-mouth six-point pocket chart	○	○	○	○	○	○	○
Intraoral radiographs	○	○	○	○	○	○	○
Panoramic radiographs	○	○	○	○	○	○	○
CT/CBCT	○	○	○	○	○	○	○

24. How many times a year should a patient with full arch, fixed prosthesis on implants receive professional hygiene care? *

In absence of potential risk factors for biological and/or technical complications:

The value must be a number

25. How many times a year should a patient with full arch, fixed prosthesis on implants receive professional hygiene care? *

In presence of potential risk factors for biological and/or technical complications:

The value must be a number

26. For screw-retained full arch, fixed prosthesis on implants, how often should it be removed for hygiene? *

○ Never

○ Only in case of peri-implantitis

○ Regularly

27. If "regularly", please indicate at which interval (in months) *

The value must be a number

28. In case you remove it, do you then change the prosthetic screws? *

○ Yes

○ No

29. For bar-supported overdenture, how often should the bar be removed for hygiene? *

- Never
- Only in case of peri-implantitis
- Regularly

30. If regularly, please indicate at which interval (in months) *

The value must be a number

31. In case you remove it, do you then change the prosthetic screws? *

- Yes
- No

32. For a full arch, fixed prosthesis on implants, do you recommend the following home care devices and products to patients? *

Per each answer, please indicate your level of agreement with a score from "strongly disagree" to "strongly agree" ***Make sure to slide the table all the way to the right to see all score levels, including "strongly agree."***

	strongly disagree	disagree	somewhat disagree	neither agree or disagree	somewhat agree	agree	strongly agree
Manual toothbrush							
Electric toothbrush							
Toothpaste							
Mouthwash							
Interdental brush							
Dental floss							
Oral irrigator							

33. In absence of parafunctions, in case of maxillary full arch, fixed prosthesis on implants do you recommend an occlusal guard? *

- Always
- Yes, in selected cases
- Never

34. If "yes", depending on ... *

Select at least one from the four options below

- [ ] Material of the restoration
- [ ] Type of antagonist
- [ ] Number of implants
- [ ] Implant length

35. As regards soft tissue management, in which circumstances the clinician deems soft tissue augmentation to be necessary to establish keratinized mucosa around dental implants? *

Per each answer, please indicate your level of agreement with a score from "strongly disagree" to "strongly agree" ***Make sure to slide the table all the way to the right to see all score levels, including "strongly agree."***

	strongly disagree	disagree	somewhat disagree	neither agree or disagree	somewhat agree	agree	strongly agree
Lack of keratinized mucosa	○	○	○	○	○	○	○
Thin keratinized mucosa	○	○	○	○	○	○	○
Keratinized mucosa width<2mm	○	○	○	○	○	○	○
Lack of vestibule	○	○	○	○	○	○	○
Anterior maxilla	○	○	○	○	○	○	○
Posterior maxilla	○	○	○	○	○	○	○
Removable restorations	○	○	○	○	○	○	○
Fixed restorations	○	○	○	○	○	○	○

36. In case of maxillary full-arch rehabilitation with dental implants, do the following factors affect the overall patient satisfaction in the short term? *

Per each answer, please indicate your level of agreement with a score from "strongly disagree" to "strongly agree" ***Make sure to slide the table all the way to the right to see all score levels, including "strongly agree."***

	strongly disagree	disagree	somewhat disagree	neither agree or disagree	somewhat agree	agree	strongly agree
Aesthetics	◯	◯	◯	◯	◯	◯	◯
Chewing function	◯	◯	◯	◯	◯	◯	◯
Type of definitive prosthesis (i.e. fixed vs removable)	◯	◯	◯	◯	◯	◯	◯
Phonetics	◯	◯	◯	◯	◯	◯	◯
Easy of cleaning	◯	◯	◯	◯	◯	◯	◯
Preoperative dental status	◯	◯	◯	◯	◯	◯	◯
Discomfort with the surgery	◯	◯	◯	◯	◯	◯	◯
Invasiveness of the procedure	◯	◯	◯	◯	◯	◯	◯
Number of surgeries	◯	◯	◯	◯	◯	◯	◯
Complications	◯	◯	◯	◯	◯	◯	◯
Overall duration of the treatment	◯	◯	◯	◯	◯	◯	◯
Duration of the surgery	◯	◯	◯	◯	◯	◯	◯
Loading protocol	◯	◯	◯	◯	◯	◯	◯
Pain	◯	◯	◯	◯	◯	◯	◯
Need for medications	◯	◯	◯	◯	◯	◯	◯
Costs	◯	◯	◯	◯	◯	◯	◯

37. When you choose a procedure, do you base your decision on? *

◯ Ease of treatment

◯ Evidence

◯ Both

38. How important is the difficulty of the procedure when you choose one? *

Please indicate your level of agreement with a score from "strongly disagree" to "strongly agree" ***Make sure to slide the table all the way to the right to see all score levels, including "strongly agree."***

	strongly disagree	disagree	somewhat disagree	neither agree or disagree	somewhat agree	agree	strongly agree
Agreement	◯	◯	◯	◯	◯	◯	◯

39. In future studies on maxillary full-arch rehabilitation with dental implants, do you consider relevant the following patient reported outcome measures (PROMs)? *

Per each answer, please indicate your level of agreement with a score from "strongly disagree" to "strongly agree" ***Make sure to slide the table all the way to the right to see all score levels, including "strongly agree."***

	strongly disagree	disagree	somewhat disagree	neither agree or disagree	somewhat agree	agree	strongly agree
Quality of masticatory function questionnaire (QMFQ)	◯	◯	◯	◯	◯	◯	◯
Oral health impact profile for edentulous people (OHIP-EDENT)	◯	◯	◯	◯	◯	◯	◯
Visual analogue scale (VAS) for satisfaction	◯	◯	◯	◯	◯	◯	◯
Numerical rating scale (NRS) for satisfaction	◯	◯	◯	◯	◯	◯	◯
Chewing ability questionnaire	◯	◯	◯	◯	◯	◯	◯
Denture satisfaction questionnaire	◯	◯	◯	◯	◯	◯	◯
Micro aesthetics questionnaire (from smile close up photos)	◯	◯	◯	◯	◯	◯	◯
Macro aesthetics questionnaire (from smile portrait photos)	◯	◯	◯	◯	◯	◯	◯

40. In future studies on maxillary full-arch rehabilitation with dental implants, do you consider relevant the following clinician- (CROM) reported outcome measures? *

Per each answer, please indicate your level of agreement with a score from "strongly disagree" to "strongly agree" ***Make sure to slide the table all the way to the right to see all score levels, including "strongly agree."***

	strongly disagree	disagree	somewhat disagree	neither agree or disagree	somewhat agree	agree	strongly agree
Implant survival	○	○	○	○	○	○	○
Vertical bone height assessed on CBCT	○	○	○	○	○	○	○
Surgical complications	○	○	○	○	○	○	○
Prosthetic complications	○	○	○	○	○	○	○
Marginal bone loss (MBL)	○	○	○	○	○	○	○
Resonance frequency analysis	○	○	○	○	○	○	○
Implant primary stability	○	○	○	○	○	○	○
Plaque score	○	○	○	○	○	○	○
Peri-implant mucositis and Peri-implantitis	○	○	○	○	○	○	○
Duration of the surgery	○	○	○	○	○	○	○
Ridge Width Measurement	○	○	○	○	○	○	○
Ridge Height Measurement	○	○	○	○	○	○	○
Keratinized Tissue Width	○	○	○	○	○	○	○
Clinician's Assessment of Treatment Success	○	○	○	○	○	○	○
Recommendations for Future Procedures	○	○	○	○	○	○	○
Surgery Difficulty	○	○	○	○	○	○	○

This content is neither created nor endorsed by Microsoft. The data you submit will be sent to the form owner.

Microsoft Forms
