# Supplementary material for: 1st Global Consensus for Clinical Guidelines for the Rehabilitation of the Edentulous Maxilla: A Single‐Round Survey on Standard, Short, and Zygomatic Implant‐Supported Prostheses
Source: Clin Oral Implants Res. 2026 Feb 24;37(Suppl 30):S135–54. doi: 10.1111/clr.70015 (PMC12930133; doi:10.1111/clr.70015)
Supplement: Supplementary file 2 — Appendix S2: clr70015‐sup‐0002‐Supinfo02.docx. [file CLR-37-S135-s001.docx]

**SUPPLEMENTARY FIGURES**

For each 7-point Likert scale question, a graph was created reporting medians and interquartile ranges (IQR).

**Suppl. Figure 1. To what extent do you agree with the following statements regarding zygomatic implants?**

**Suppl. Figure 2. To what extent do you agree with the following statements regarding short implants as compared with standard implants and sinus lift/bone grafting?**


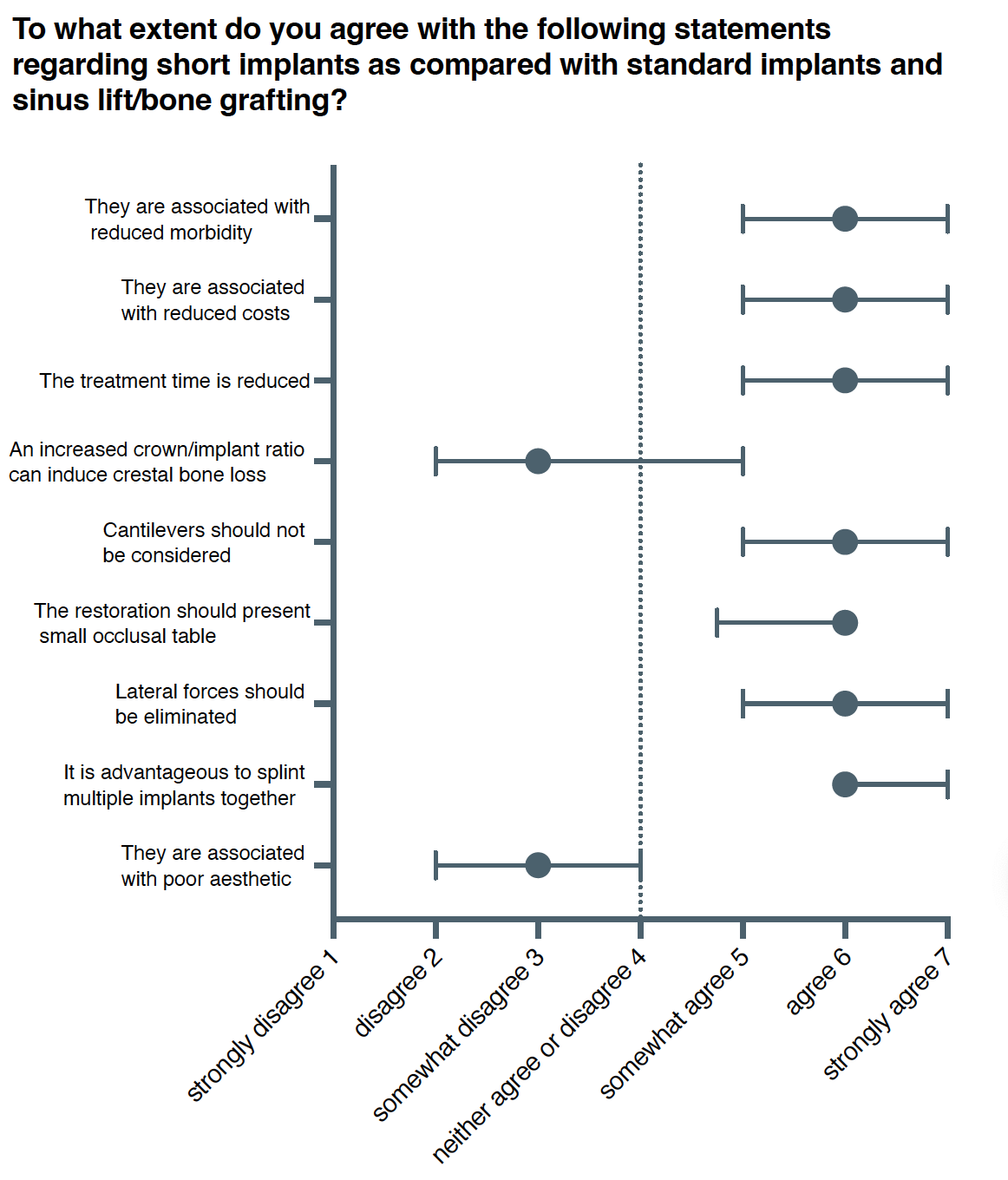


**Suppl. Figure 3. Which tool(s) should be routinely used for case study of maxillary full-arch rehabilitation with implants?**

**Suppl. Figure 4. Prior to implant surgery in the posterior maxilla, in which cases do you consider necessary to check the status of the maxillary sinuses (e.g. presence of septa, ostium patency, mucous membrane thickening, sinus pathologies)?**

(*) combined use of standard implants not excluded

**Suppl. Figure 5. In case of multiple implant placements in fully edentulous maxilla, should freehand surgery be preferred over static/dynamic guided surgery?**

(*) combined use of standard implants not excluded

**Suppl. Figure 6. As regards soft tissue management, in which circumstances the clinician deems soft tissue augmentation to be necessary to establish keratinized mucosa around dental implants?**

**Suppl. Figure 7. In case of multiple implant placements in fully edentulous maxilla, which are the factors that determine the decision to proceed with immediate implant loading?**

**Suppl. Figure 8. For maxillary full-arch rehabilitation with implants, is delayed loading to be preferred over immediate loading?**

(*) combined use of standard implants not excluded

**Suppl. Figure 9. How important is the difficulty of the procedure when you choose one?**

**Suppl. Figure 10. In absence of complications, are the following procedures justified at least once a year in the long-term follow-up?**

**Suppl. Figure 11. For a full arch, fixed prosthesis on implants, do you recommend the following home care devices and products to patients?**

**Suppl. Figure 12. In case of maxillary full-arch rehabilitation with dental implants, do the following factors affect the overall patient satisfaction in the short term?**

**Suppl. Figure 13. In future studies on maxillary full-arch rehabilitation with dental implants, do you consider relevant the following patient-reported outcome measures (PROMs)?**

**Suppl. Figure 14. In future studies on maxillary full-arch rehabilitation with dental implants, do you consider relevant the following clinician-reported outcome measures (ClinROMs)?**
